# Assessment of the variational quantum eigensolver (VQE) for bond dissociation of H$_{2}$, H${_3^+}$, and CH$_{5}^{+}$

**DOI:** 10.1007/s00894-026-06951-w

**Published:** 2026-09-25

**Authors:** Amanda Marques de Lima, Eivson Darlivam Rodrigues de Aguiar Silva, Erico Souza Teixeira, Ricardo Luiz Longo

**Affiliations:** 1 Departamento de Química Fundamental, Universidade Federal de Pernambuco, Recife, PE 50670-901 Brazil; 2 Centro de Excelência em Computação Quântica, Venturus Centro de Inovação Tecnológica, Estrada Giuseppina Vianelli di Napolli, 1185, Campinas, SP 13086-530 Brazil

**Keywords:** VQE, Quantum computational chemistry, Ground state energy, Quantum simulator, Dissociation profile

## Abstract

**Context:**

The variational quantum eigensolver (VQE) is a quantum-classical algorithm for estimating molecular ground-state energies. However, the accuracy depends on the choice of *ansatz*, active space, and initial variational parameters. Hence, it is relevant to perform assessments of VQE. The dissociation profiles of H$_2$, H$_3^+$, and CH$_5^+$ were chosen for these assessments. For H$_2$ and H$_3^+$, the UCCSD, GateFabric, and *k*-UpCCGSD *ansätze* reproduced the dissociation profiles with high accuracy. For CH$_5^+$, GateFabric and *k*-UpCCGSD provided the best compromise between accuracy and scalability. Hardware-efficient *ansätze* (HEA), particularly HEA-A and HEA-B, produced significant deviations from the reference energies. The results also demonstrate that larger numbers of parameters or logic gates do not necessarily improve VQE performance. Furthermore, some *ansätze*, especially GRSD and EHA, showed sensitivity to the initialization of the parameters.

**Methods:**

Reference geometries and energies were obtained at the CISD/STO-3 G level using Gaussian 09. VQE simulations were performed with the PennyLane library employing the Jordan-Wigner mapping. The *ansätze* evaluated were UCCSD, *k*-UpCCGSD, GateFabric, GRSD, HEA-A, HEA-B, HEA-C, and Entanglement-variational Hardware-efficient *Ansatz* (EHA). Dissociation profiles were generated by varying interatomic distances and angular coordinates for H$_2$ and H$_3^+$, as well as the C-H$_2$ dissociation coordinate for CH$_5^+$. Active-space ranging from 2/2 to 8/8 electrons/orbitals was employed for CH$_5^+$. Variational parameters were optimized using stochastic gradient descent with a convergence threshold of $1 \times 10^{-6}$ E$_h$. The VQE energies were obtained from averages over multiple runs using both zeros and random parameter initializations.

**Supplementary Information:**

The online version contains supplementary material available at https://doi.org/10.1007/s00894-026-06951-w.

## Introduction

The Schrödinger equation provides the foundation for predicting and understanding energetic and all other molecular properties [10, 48, 56]. However, solving it exactly for many-electron systems is unfeasible, prompting the development of approximate methods. While successful, these methods are designed for classical computers, where computational cost significantly increases with system size and complexity [18]. For example, capturing electronic correlations in transition metal clusters like FeMoco (cofactor FeMo) [39, 45, 53] requires advanced methods like the Complete Active Space Self-Consistent Field (CASSCF) [44], but its computational complexity scales factorially, *O*(*N*!), with the number of electrons *N* [25].

Quantum algorithms have attracted attention for simulating fundamental electronic states because they can scale more favorably than approaches used in classical computers, avoiding factorial or exponential costs [4, 19]. Several quantum algorithms have been proposed for this purpose [1, 18, 23]. One of the most relevant is Quantum Phase Estimation (QPE) [35], a fully quantum algorithm that extracts eigenvalues of a unitary operator using the Quantum Fourier Transform (QFT) [20]. However, QPE often requires millions of qubits and gates, exceeding the capabilities of current noisy intermediate-scale quantum devices (NISQ) [24, 51].

A more practical alternative for the NISQ era is the variational quantum eigensolver (VQE) [49]. The VQE mitigates scalability challenges by requiring fewer qubits than the QPE and the transfer the step of with parameter optimization to a classical computer, making it more resilient to decoherence and noise [1, 15, 36]. This resilience has enabled its application in calculating molecular and energetic properties with reasonable accuracy [33, 41, 66]. Due to its potential, the VQE has attracted significant attention, with ongoing research focused on improving its efficiency, accuracy, and scalability for larger and more complex systems [7, 9].

A potential application of VQE is to investigate molecular dissociation, which provides the basis for understanding macroscopic chemical behavior [5, 62]. Dissociation involves forming and breaking bonds, which are the foundation of most chemical transformations [12]. Notable examples of dissociation processes include the bond breaking of H$_2$ into hydrogen atoms (symmetric breaking) or into H$^-$ and H$^+$ ions (asymmetric breaking). The dissociation of H$_3^+$ can produce H$_2$ + H$^+$ or two hydrogen atoms and a proton [37]. Another example is the dissociation of protonated methane (CH$_5^+$) through the loss of an H$_2$ molecule [41, 47].

Notice that even the dissociation of simple systems such as H$_2$, H$_3^+$, and CH$_{5}^{+}$ required robust methods that can account for complex electronic correlation effects. Within VQE, these effects depend on the choice of the *ansatz* (e.g., chemically inspired or hardware efficient), the active space employed to minimize the number of qubits required, and the parameters used to initialize the quantum circuit. Hence, it is relevant to perform assessments of VQE by comparing to some reference for selected systems. In this context, the present work employs VQE with different ansätze (e.g., UCCSD, *k*-UpCCGSD, GRSD, GateFabric, HEA-A, HEA-B, HEA-C, and EHA), active spaces with n-electrons/n-orbitals (e.g., 2/2 up to 8/8), and initial parameters (e.g., zero or average over random values) to calculate the dissociation energy profiles of H$_2$, H$_3^+$, and CH$_{5}^{+}$) and compare to CISD results. The remainder of the article is organized as follows: the “Methods” section will present some principles of VQE and the techniques used in this work, as well as the methods used in the classical computer and the quantum simulator. In the “Results and discussion” section, the results obtained for H$_2$, H$_3^+$, and, subsequently, for CH$_{5}^{+}$ will be presented and discussed, followed by some concluding remarks.

## Methods

### Variational quantum eigensolver

Variational quantum algorithms (VQA) are hybrid quantum-classical algorithms with adjustable parameters [1, 52]. VQE algorithm determines the ground-state energy of molecules by minimizing the expectation value of the Hamiltonian, $\hat{H}$. If $|\psi _0\rangle$ is the ground state, then

1 $$\begin{aligned} \hat{H} | {\psi _0} \rangle = E_0 |\psi _0 \rangle , \end{aligned}$$

where $E_0$ is the ground-state energy [59].

**Fig. 1:**
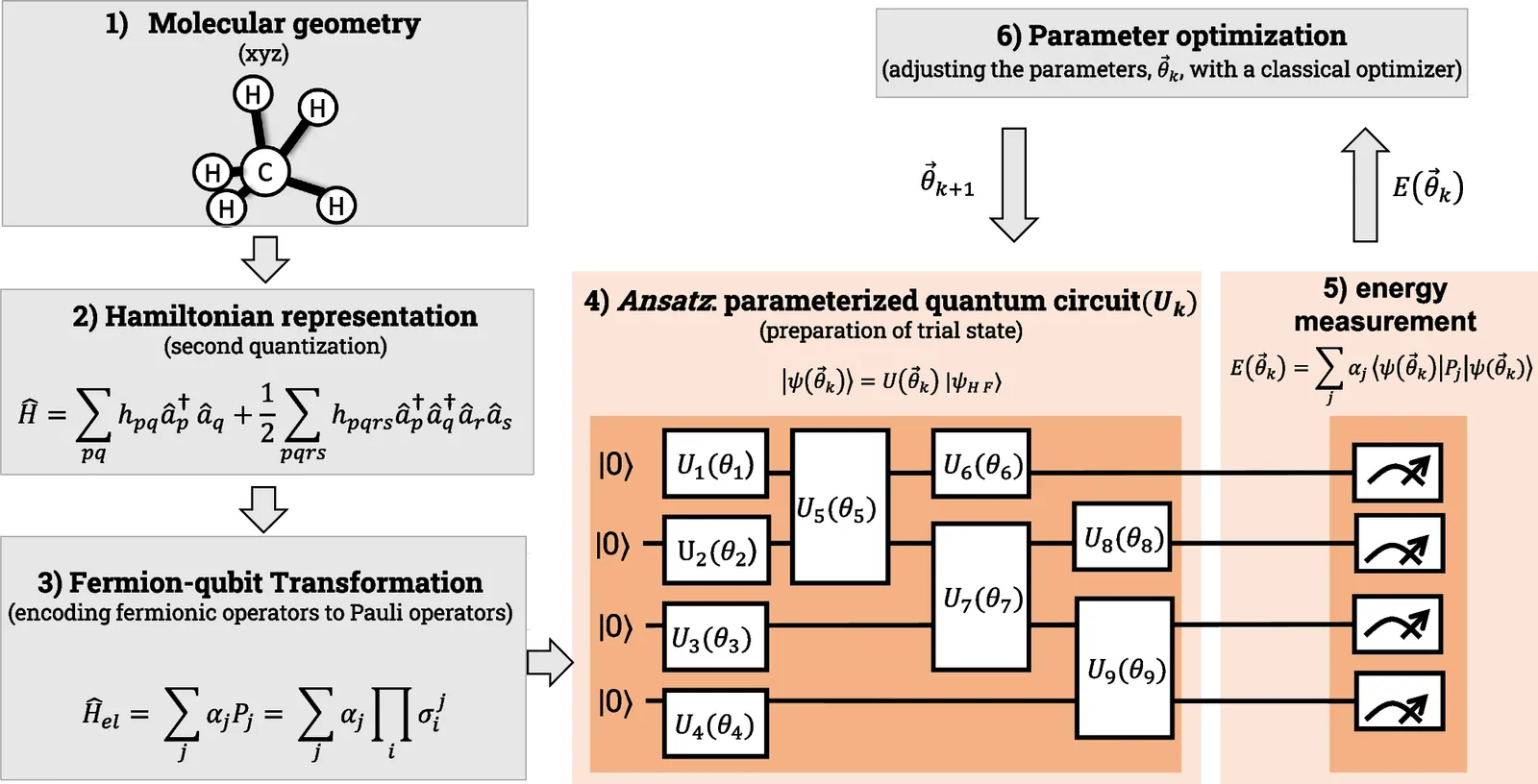
Schematic of the VQE adapted from Fedorov et al. [24]

In more detail, as shown in Fig. 1, the VQE algorithm begins with the representation of the molecular system through its fermionic Hamiltonian in the second-quantization formalism [24, 70]:

2 $$\begin{aligned} \hat{H} = \sum _{pq} h_{pq} \hat{a}_p^\dagger \hat{a}_q + \frac{1}{2} \sum _{pqrs} h_{pqrs} \hat{a}_p^\dagger \hat{a}_q^\dagger \hat{a}_r \hat{a}_s, \end{aligned}$$

where $\hat{a}_p^\dagger$ and $\hat{a}_q$ are the fermionic creation and annihilation operators, respectively, while $h_{pq}$ and $h_{pqrs}$ denote the one- and two-electron integrals, respectively.

Next, the operator $\hat{H}$ is mapped onto a qubit Hamiltonian [40] using mappings such as Jordan-Wigner [8] or Bravyi-Kitaev [14]. In the Jordan-Wigner mapping, occupied and unoccupied spin-orbitals are represented by the qubit states |1⟩ and |0⟩, respectively [64]. As a result, $\hat{H}$ is rewritten as a linear combination of Pauli strings [24]:

3 $$\begin{aligned} \hat{H}_{el} = \sum _{j} \alpha _{j} P_{j} = \sum _{j} \alpha _{j} \prod _{i} \sigma ^{j}_{i}, \end{aligned}$$

where $P_j$ denotes tensor products of Pauli operators, *i* specifies the qubit on which each operator acts, *j* labels the Hamiltonian terms, and $\alpha _j$ are real coefficients. Thus, the occupation of each spin-orbital is stored locally in a qubit, whereas the fermionic parity information is encoded in Pauli strings whose support may scale as $\mathcal {O}(N)$ with the number of spin-orbitals.

The Bravyi-Kitaev transformation provides an alternative mapping in which occupation and parity information are distributed among the qubits, reducing the maximum support of the corresponding Pauli strings to $\mathcal {O}(\log N)$ [14, 64]. This reduction in operator locality may decrease the number of one- and two-qubit gates required for circuit implementation, although the actual savings depend on the circuit decomposition, orbital ordering, and hardware connectivity [64]. This advantage may become particularly relevant for larger molecular systems and basis sets.

Both the Jordan-Wigner and Bravyi-Kitaev transformations are exact fermion-to-qubit mappings that preserve the canonical anticommutation relations and, consequently, the energy spectrum of the original fermionic Hamiltonian [14, 57]. Thus, the exact ground-state energy is independent of the selected mapping, although practical differences may arise from circuit structure, gate decomposition, optimization, and hardware noise. Because this work focuses on comparing the performance of different *ansätze* for yielding molecular energies under a common protocol, only the Jordan-Wigner transformation was employed. A comparison with Bravyi-Kitaev for larger active spaces and more flexible basis sets is an ongoing investigation that will be reported in due time.

After defining the qubit representation of the Hamiltonian, $\hat{H}_{el}$, a parameterized quantum circuit, or *ansatz*, $|\psi (\boldsymbol{\theta })\rangle$, is prepared, where $\boldsymbol{\theta }$ are the variational parameters. This trial wave function is then executed on the quantum device to evaluate the expectation value of $\hat{H}_{el}$, which defines the standard VQE cost function:

4 $$\begin{aligned} C_{VQE}(\boldsymbol{\theta }) = \langle \psi (\boldsymbol{\theta }) | \hat{H}_{el} | \psi (\boldsymbol{\theta }) \rangle . \end{aligned}$$

According to the variational principle, this value provides an upper bound to the ground-state energy, i.e., $C_{VQE}(\boldsymbol{\theta }) \ge E_0$. In practice, evaluating (4) requires running the circuit and measuring the qubits to estimate the expectation values of the Pauli strings that constitute $\hat{H}_{el}$. The resulting energy is passed to a classical optimizer, which updates the variational parameters $\boldsymbol{\theta }$ to minimize $C_{VQE}(\boldsymbol{\theta })$. This quantum-classical loop is repeated until a convergence criterion is reached. Then, the optimized parameters provide an approximation to the ground-state wave function and its corresponding energy [24, 46].

VQE faces several challenges, including the use of large *ansätze* and the need for classical optimization with numerous variational parameters, generating convergence difficulties, causing gradients to even disappear, trapping the optimization process [27, 42, 65]. Furthermore, the performance of the algorithm depends on the quality and flexibility of the *ansatz*, as well as the initial parameters [24]. Therefore, finding suitable *ansätze* and initial parameters for diverse molecular systems is crucial [42].

**Fig. 2:**
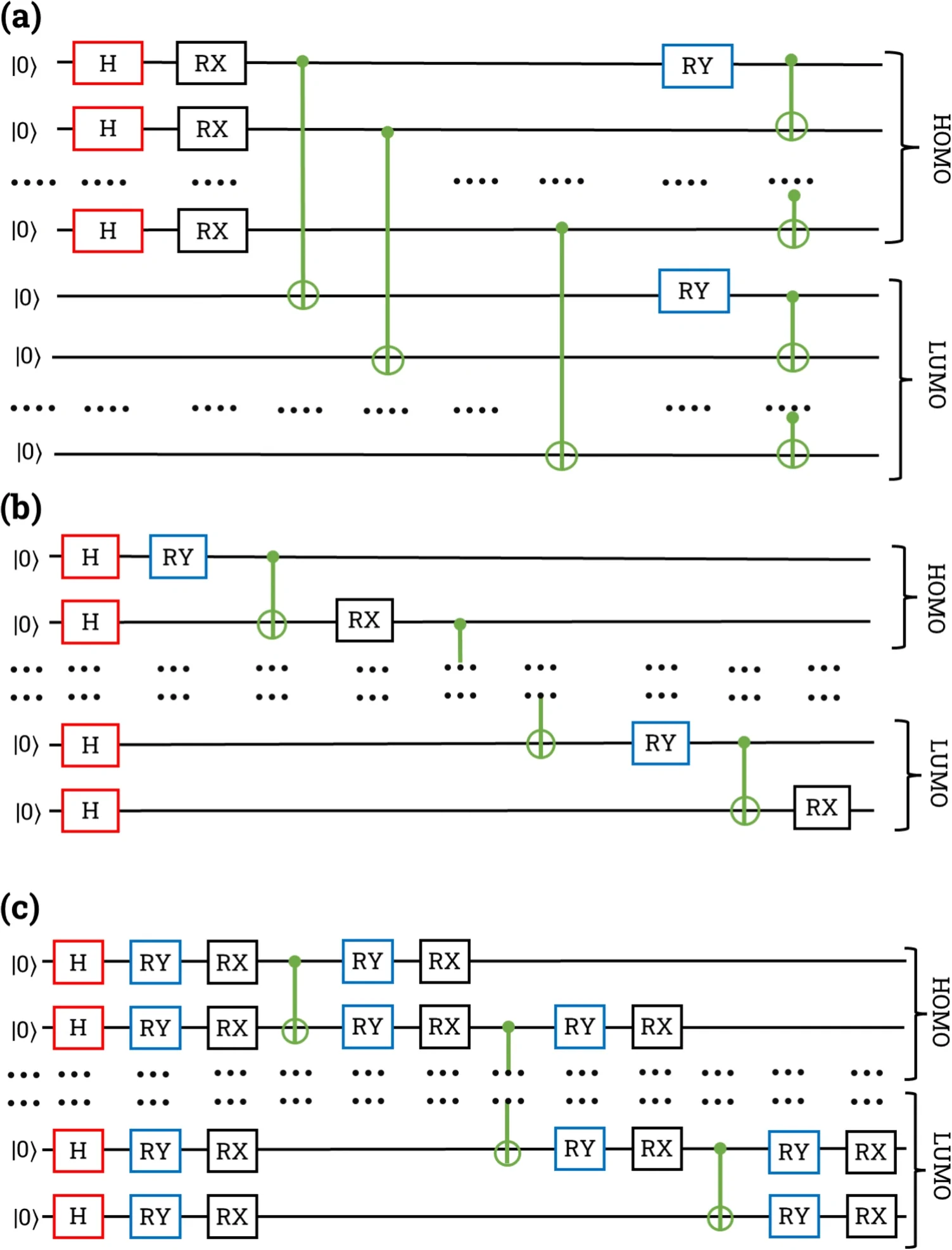
Hardware efficient circuits developed by Sennane et al. [58]. **a** HEA-A, **b** HEA-B, and **c** HEA-C. All RY and RX ports are parameterized by an angle θ. The circuit depth is equal to 1

### Ansatz

The choice of *ansatz* defines the expressiveness and training capacity of the algorithm, as it establishes the search space covered during optimization [27]. Selecting an *ansatz* that balances efficiency and precision is essential, as a simple *ansatz* may not capture important correlations, while a complex one may be computationally expensive [46, 66]. There are several ways to construct *ansatz*, which can, for example, be inspired by chemistry, i.e., quantum circuits whose logic gates simulate single and double electronic excitations to obtain the electronic correlation energy [24]. Circuits can also be designed to improve the efficiency of the hardware used, usually called hardware-efficient *ansatz* (HEAs) [34].

The most widely used chemically inspired *ansatz* is the truncated Unitary Coupled-Cluster for Single and Double Excitations (UCCSD) [54], as it can effectively capture correlation effects. However, this *ansatz* may be impractical to use in large systems [54].

The k-Unitary Pair Coupled-Cluster Generalized Singles and Doubles (*k*-UpCCGSD) [38] was created to overcome the demands of UCCSD. This circuit uses *k* exponential products of pair-coupled-cluster (pCC) double excitation operators. The pCC causes double excitations to contain only those two-body excitations of a pair of electrons from one orbital to another [38]. Furthermore, it uses generalized single and double excitation operators, which do not distinguish between occupied and unoccupied orbitals [38].

**Fig. 3:**
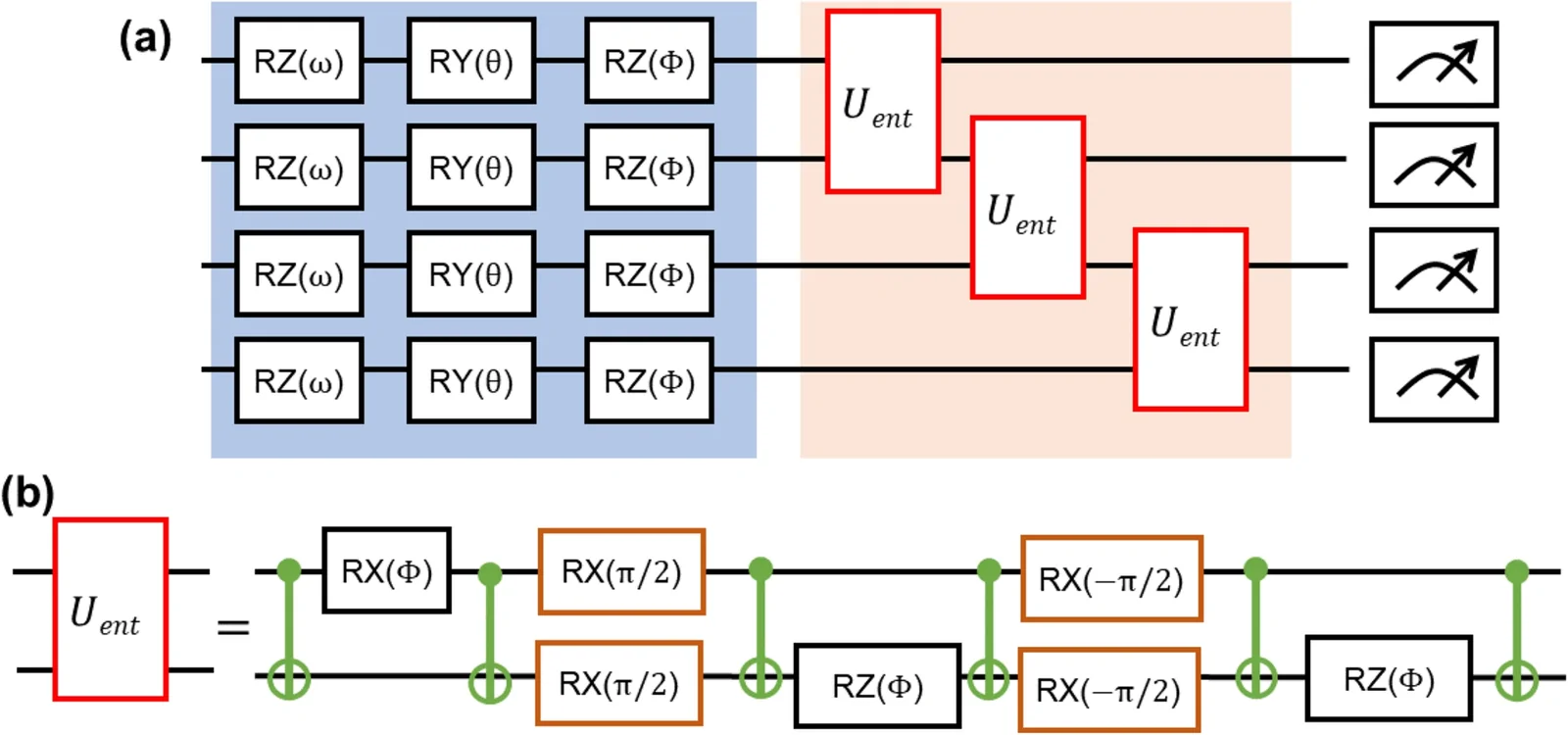
Entanglement-variational Hardware-efficient *ansatz* (EHA) developed by Wang et al. [66]. **a** Quantum circuit. **b** Each $U_{ent}$ variational entangler is composed of XX, YY, and ZZ gates in series. The variational parameters of tangles are different. The circuit depth is equal to 1

Another chemistry-inspired *ansatz* is the Givens rotation with single and double excitations (GRSD) [66]. The Givens rotation gates used in this circuit satisfy the principal symmetries of fermionic systems; therefore, they are used to ensure that spin conservation is respected in a quantum circuit [3].

GateFabric [2] is a chemistry-based and HEA-based *ansatz*. It utilizes Givens rotations, with double excitations between neighboring qubits, in addition to adding fermionic SWAP gates. This gate swaps $|01\rangle \Leftrightarrow |10\rangle$ and adds a factor of -1 when both qubits are occupied, reflecting the antisymmetry of fermions [2]. This approach is both local (the gates act only on a limited number of neighboring qubits) and expressive (it represents quantum states and preserves certain invariant quantum numbers) [2].

Unlike chemically inspired *ansätze*, which are constructed from fermionic excitation operators, HEA are heuristic and generally problem-agnostic circuits [46]. Their architectures are designed primarily to reduce circuit depth and two-qubit-gate overhead by employing shallow layers of parameterized single-qubit rotations and entangling gates compatible with the connectivity and native gate set of quantum hardware [34, 46, 69]. This reduced circuit cost makes HEA attractive for NISQ devices, but their construction does not explicitly incorporate the symmetries of the molecular Hamiltonian [34]. In particular, they do not generally preserve the electron-number operator $\hat{N}$ or the total-spin operator $\hat{S}^2$ by construction [66].

Numerous HEAs have been proposed in the literature. Among them, Sennane et al. [58] introduced the HEA-A, HEA-B, and HEA-C circuits shown in Fig. 2 to calculate the ground-state energy of benzene at distorted geometries. HEA-A applies Hadamard gates to the first $N_{\textrm{qubits}}/2$ qubits, followed by CNOT entangling gates and parameterized single-qubit rotations. In the Jordan-Wigner occupation representation, the Hadamard transforms a qubit into a superposition of occupied and unoccupied states. The subsequent CNOT gates couple the qubits associated with the initially occupied and virtual orbital sectors. HEA-B employs parameterized $R_X$ and $R_Y$ rotations to provide additional control over the occupation amplitudes, whereas HEA-C contains a larger number of single-qubit rotations, including additional $R_X$ and $R_Y$ gates following the entangling operations [58]. Because the Hadamard, $R_X$, and $R_Y$ gates mix the computational states |0⟩ and |1⟩, HEA-A, HEA-B, and HEA-C can generate superpositions containing determinants with electron numbers different from the target value. Their generic gate structure may also mix states belonging to different spin sectors.

Wang et al. [66] developed a HEA called Entanglement-variational Hardware-efficient (EHA) (Fig. 3). The EHA incorporates RX and RY rotations followed by adjustable entanglement gates, U$_{ent}$, which differentiates the EHA from existing hardware-efficient entanglement approaches such as HEA-A, -B, and -C. Thus, while many hardware-efficient entanglement approaches use fixed entanglement gates, the EHA introduces the idea that these gates can be variationally adjusted [66].

**Fig. 4:**
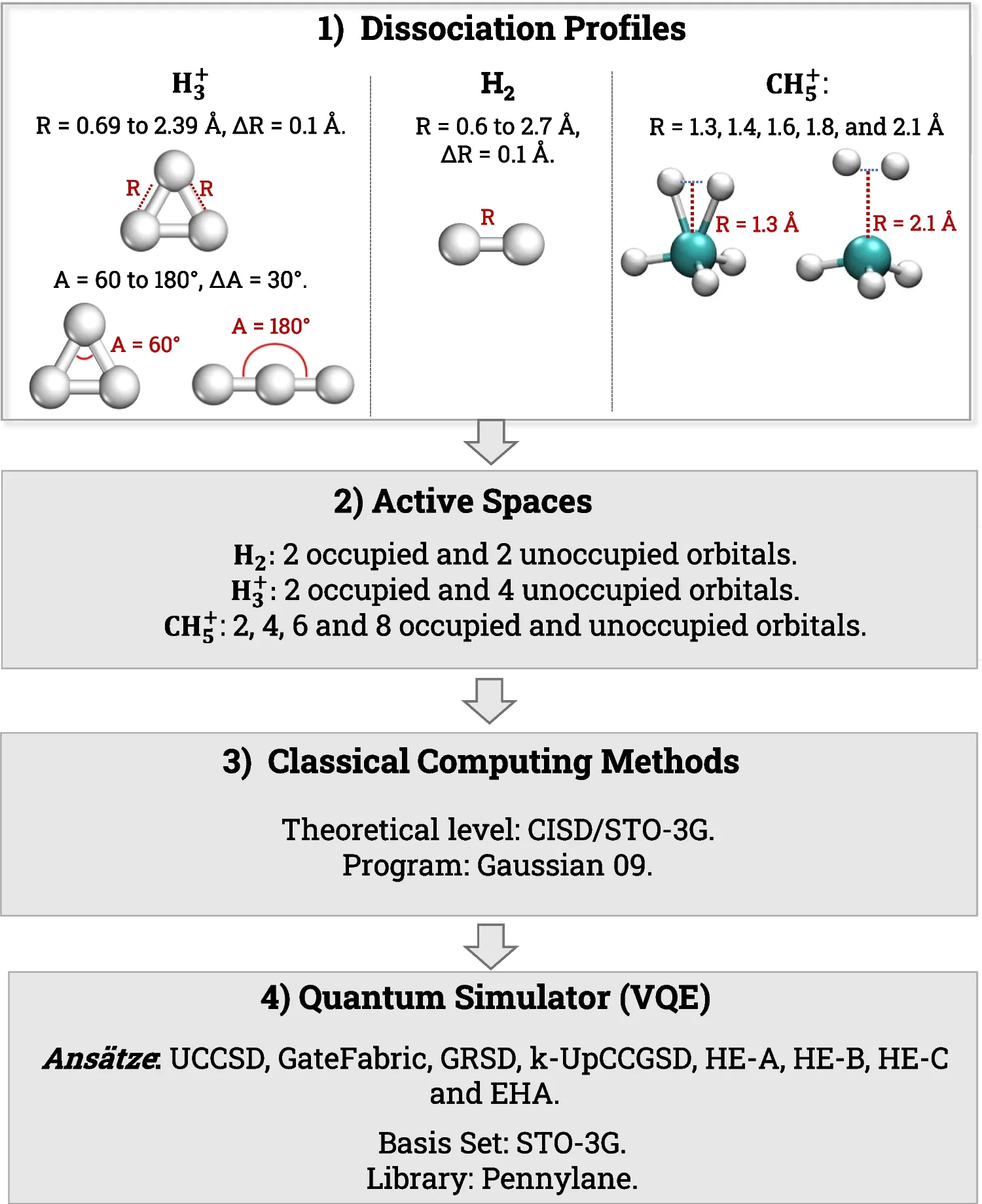
Flowchart carried out in this work to obtain the ground-state energy of H$_2$, H$_3^+$, and CH$_{5}^{+}$ using classical computer and quantum computer simulator (VQE with different *ansätze*)

To reduce the contribution of states with an incorrect number of electrons in HEA, the cost function was supplemented with the particle-number penalty [66]:

5 $$\begin{aligned} C(\theta ) = \langle \psi (\theta ) | H_{el} | \psi (\theta ) \rangle + \beta \left[ \langle \psi (\theta ) | N | \psi (\theta ) \rangle - ac \right] ^2, \end{aligned}$$

where $H_{el}$ is the Hamiltonian (3), β is the penalty hyperparameter which was fixed at 2, *ac* is the number of active electrons, and $N = \sum _{\alpha } \hat{c}_\alpha ^{\dagger } \hat{c}_\alpha$, denotes the number of particles, with $\hat{c}_\alpha ^{\dagger }$ and $\hat{c}_\alpha$ being the particle creation and annihilation operators, respectively. This term penalizes deviations from the expected value $\langle \hat{N}\rangle$ relative to the target number of active electrons. However, this expected value constraint does not project the wave function onto a subspace with a fixed number of particles. Therefore, an optimized state may still contain non-zero contributions from sectors with different numbers of electrons, even though, on average across all states, the particle number is close to the number of active electrons.

### Case studies

Figure 4 shows the flowchart of the steps performed in this work. The dissociation profiles of H$_2$, H$_3^+$, and CH$_5^+$ were obtained by varying the length or angle associated with the dissociation coordinate as depicted in Fig. 4. For H$_2$, the interatomic distance was varied from 0.6 to 2.7 Å, in 0.1 Å intervals. H$_3^+$ was dissociated into H$_2$ and H$^+$ by varying the distance from 0.69 to 2.39  Å, in 0.1 Å intervals. Analyses were also performed as a function of the angle between the three H atoms of H$_3^+$, starting from the equilibrium angle ($0^{\circ }$) to the linear geometry of H$_3^+$ ($180^{\circ }$), in $30^{\circ }$ increments.

CH$_5^+$ is a prototypical fluxional molecule, that is, a non-rigid molecular system that undergoes rapid, large-amplitude rearrangements among several nearly equivalent structures. Consequently, the hydrogen atoms can continuously exchange their positions around the carbon center, a phenomenon known as hydrogen scrambling [32, 67]. Thus, CH$_5^+$ cannot be adequately described by a single rigid geometry or by a permanently defined set of C-H bonds.

For CH$_5^+$, the dissociation coordinate was defined as the distance between the carbon atom and the center of mass of the outgoing H$_2$ fragment, which is approximately 1.3 Å in the equilibrium region. Because a complete treatment of the fluxional motion and hydrogen scrambling would require the simultaneous description of several coupled nuclear coordinates, the scan along the C–H$_2$ coordinate represents a simplified model of the dissociation process. At each point of the scan, the distance between the carbon atom and the center of mass of H$_2$ was constrained, while the remaining bond lengths and bond angles were optimized. The dissociation intermediates were obtained by setting this coordinate to 1.4, 1.6, 1.8, and 2.1 Å (Fig. 4). These distances were chosen to provide a comprehensive representation of the binding and dissociation regions [47]. Despite its simplified one-dimensional description, this dissociation coordinate provides a challenging and informative assessment of VQE and *ansätze*. As the C–H$_2$ distance increases, the electronic structure undergoes reorganization and acquires some multireference behavior, requiring the quantum circuits to describe strong electronic correlation and near-degenerate electronic configurations along the dissociation pathway.

The optimization of the dissociating structure and the energies were obtained using Configuration Interaction Singles and Doubles (CISD) [31], with the STO-3 G basis set [21, 30] and using the Gaussian 09 program [26] in a classical computer.

The STO-3 G basis set was selected to keep the quantum simulations computationally feasible. In this minimal basis, each contracted atomic orbital is represented by three primitive Gaussian functions [22]. The resulting reduction in the number of molecular orbitals decreases the number of qubits required under standard fermion-to-qubit mappings and typically reduces the number of variational parameters and quantum gates in the corresponding circuits. This computational advantage is obtained at the cost of limited orbital flexibility and reduced quantitative accuracy, particularly along bond-dissociation coordinates. Accordingly, the calculations performed in this work are intended to provide a consistent comparison of different *ansätze* within a common and computationally tractable electronic-structure model [41], rather than quantitatively exact predictions of molecular dissociation energies.

Within this computational framework, the interpretation of CISD as a reference depends on the number of electrons in the system. For the two-electron H$_2$ and H$_3^+$ systems, CISD is equivalent to Full Configuration Interaction (FCI) within a fixed finite orbital basis because all possible substitutions relative to a reference determinant involve, at most, double excitations [31]. However, this equivalence does not apply to CH$_5^+$, which contains ten electrons. In this case, CISD provides a truncated description of the electronic wave function because it includes only single and double excitations relative to the reference determinant. Moreover, for systems containing more than two electrons, CISD is not size-consistent; when the molecular fragments become noninteracting, the energy calculated for the entire system is not necessarily equal to the sum of the energies of the isolated fragments [6, 28]. Consequently, CISD/STO-3 G should not be regarded as an exact reference for the asymptotic dissociation limit of CH$_5^+$.

### Active spaces

In a minimal STO-3 G basis set, the H$_2$ and H$_3^+$ systems contain only two electrons distributed over four and six spin-orbitals, respectively. Under the Jordan-Wigner mapping, where each spin-orbital corresponds to one qubit [41], these simulations require four and six qubits, respectively. In contrast, CH$_5^+$ involves ten electrons and twenty spin-orbitals in the same basis set, demanding a 20-qubit representation. Simulating variational quantum circuits of this scale—particularly for *ansätze* with high gate counts and numerous parameters—becomes excessively time- and memory-consuming. Therefore, to make quantum simulations of larger molecular systems computationally feasible, reducing the number of explicitly represented spin-orbitals is mandatory.

A widely adopted strategy consists of selecting an active space. Well-established in standard electronic-structure methods such as CASCI [31], this approach partitions the molecular orbital space into inactive (core), active, and external virtual orbitals. The lower-energy core orbitals remain doubly occupied and frozen, whereas the higher-energy external virtual orbitals are entirely excluded from the correlated calculation. Consequently, only a selected subset of active electrons and active orbitals is explicitly treated by the computationally demanding method [13, 50].

This framework becomes critical when expanding to larger basis sets, such as triple-ζ. While these larger bases provide a more flexible and accurate description of the electronic structure, they cause a significant increase in the number of spatial orbitals and Hamiltonian terms, creating a severe computational bottleneck. Although truncating the orbital space may omit a fraction of the dynamic correlation, progressively increasing the active space yields a more complete wave function description at the expense of optimization complexity and computational cost. Because this is a common strategy for quantum computing in the NISQ era, it is relevant to assess the performance of the ansätze regarding the use and selection of active spaces.

**Fig. 5:**
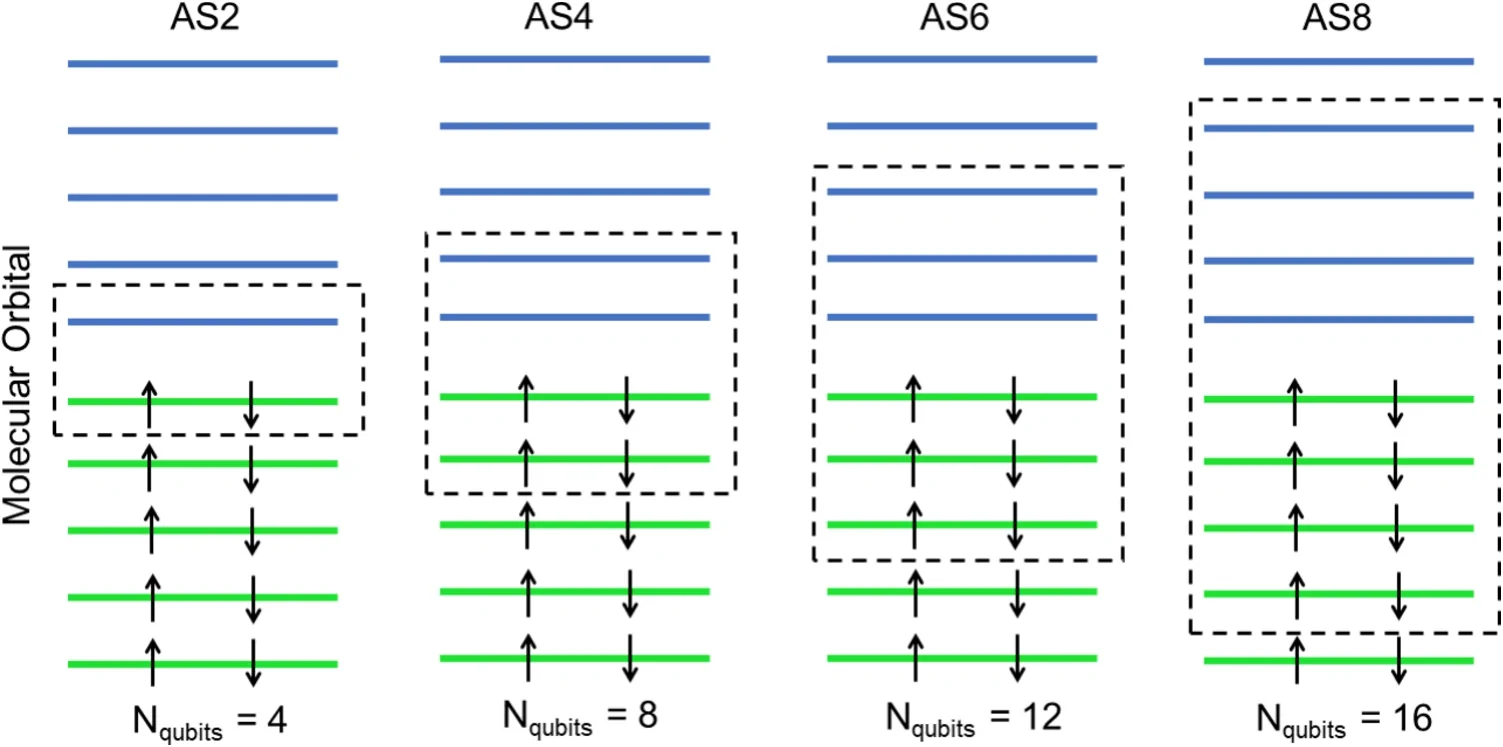
Active spaces considered for CH$_{5}^{+}$ calculations. Spin orbitals in each active space (AS) are highlighted with dashed line boxes. The AS*n* label reflects the number of occupied and unoccupied spin-orbitals considered, represented in green and blue, respectively

In this work, the active-space approximation was employed for CH$_5^+$ to balance computational feasibility with an accurate description of electronic correlation [24, 55]. The active orbitals were selected around the frontier region, centered on the highest occupied (HOMO) and lowest unoccupied (LUMO) molecular orbitals [31], as they are the most relevant for capturing electronic excitations and correlation effects [43].

Four active spaces were evaluated for CH$_5^+$ by progressively expanding the orbital window around the frontier molecular orbitals (Fig. 5). AS2 included the HOMO and LUMO, corresponding to two active electrons distributed over two spatial orbitals, four spin-orbitals, and four qubits. AS4 additionally included HOMO-1 and LUMO+1, resulting in four active electrons distributed over four spatial orbitals, eight spin-orbitals, and eight qubits. AS6 included the occupied orbitals from HOMO-2 to HOMO and the virtual orbitals from LUMO to LUMO+2, corresponding to six active electrons, six spatial orbitals, twelve spin-orbitals, and twelve qubits. Finally, AS8 included the occupied orbitals from HOMO-3 to HOMO and the virtual orbitals from LUMO to LUMO+3, resulting in eight active electrons distributed over eight spatial orbitals, sixteen spin-orbitals, and sixteen qubits.

To ensure a rigorous benchmark, reference active-space calculations were required within the exact same orbital subspaces, restricting single and double excitations to the active region while integrating the frozen core contributions into the effective Hamiltonian. Notably, while the VQE calculations were confined to these active spaces, all underlying molecular geometries were pre-optimized using the full electron and orbital complement in the STO-3 G basis set.

Despite these baseline definitions, the choice of an appropriate reference method must be carefully considered in relation to the active-space approximation commonly employed in quantum simulations. As discussed by de Lima et al. [41], VQE calculations generally require the selection of an active space to reduce the number of spin-orbitals and, consequently, the number of qubits and the complexity of the resulting circuits. Therefore, a direct comparison between an active-space VQE calculation and a Full Configuration Interaction (FCI) result in the full basis would not constitute a balanced benchmark. Whereas FCI includes excitations involving all orbitals represented in the molecular basis, the VQE calculations considered here are restricted to the selected active orbitals. Such a comparison would combine the error introduced by the active-space truncation with the representational and optimization errors of the variational circuit, preventing an unambiguous assessment of the *ansatz* [41].

For this reason, a more appropriate benchmark is obtained by performing CISD or Complete Active Space Configuration Interaction (CASCI) calculations within the same active space used by the corresponding VQE circuit. In particular, CASCI performs an exact diagonalization of the electronic Hamiltonian within the selected active space and provides a more direct and rigorous reference for evaluating active-space VQE calculations [41]. This additional comparison is especially relevant because several of the *ansätze* investigated in this work did not achieve chemical accuracy even relative to the full-basis CISD/STO-3 G reference. Thus, agreement with CISD should not be interpreted as agreement with an exact or complete treatment of the electronic structure, and comparisons with CASCI within the active space may reveal additional discrepancies that are not apparent at the full CISD/STO-3 G level.

### Details of quantum computer simulations

The VQE was implemented using the PennyLane library [11]. To use VQE, the active spaces shown in Fig. 5 were considered. The UCCSD, *k*-UpCCGSD, and GateFabric *ansätze* were tested using internal versions of Pennylane. The GRSD, HEA-A, HEA-B, HEA-C [58], and EHA [66] were constructed from the native PennyLane gates.

The depth of all circuits used is equal to one. In PennyLane, the number of GateFabric parameters is calculated as: shape=(D,L,2), where *D* is the number of GateFabric layers and L=N/(2-1), for an *N* number of qubits. In this work, *D* was considered equal to the number of electrons in the molecule. The fermion-to-qubit mapping used was Jordan-Wigner [24].

The stochastic gradient descent (SGD) optimizer was employed for all VQE calculations. It is widely accepted that gradient-based optimizers outperform non-gradient methods [29, 63]. A fixed learning rate of 0.4 was adopted for all *ansätze* as a common VQE optimization protocol [41]. By keeping both the classical optimizer and its hyperparameters unchanged throughout the calculations, the comparative analysis could focus primarily on the influence of the quantum-circuit architecture and parameter initialization, rather than on variations introduced by different classical optimization algorithms. The calculation converged when the energy difference between steps was less than $1 \times 10^{-6}$ Hartree (E$_h$). All calculations were performed on a cluster equipped with an NVIDIA GeForce GTX 980 Ti, 32 cores, and 64 GB of installed RAM.

The energies at each point of the dissociation profiles were obtained from ten independent VQE runs. Each geometry along the dissociation coordinate was optimized independently. For every geometry and *ansatz*, the first VQE run was initialized with all variational parameters set to zero, whereas the remaining nine runs were initialized with independently sampled random parameters in the interval from 0 to π/2. The optimized parameters obtained for one geometry were therefore not transferred to any neighboring point of the dissociation profile. This methodological choice was deliberate, as it allowed the intrinsic robustness of each *ansatz* with respect to parameter initialization to be assessed independently at different molecular geometries, without introducing a path-dependent bias associated with the successful convergence of a preceding point. An alternative strategy commonly employed is parameter tracking [17], in which the optimized parameters obtained for a geometry are used as an initial guess for a neighboring geometry along the potential energy curve. Because similar molecular geometries usually have similar electronic structures, this procedure can accelerate convergence and promote smoother energy profiles. However, it can obscure the dependence and robustness of an ansatz on its initial parameters. Therefore, independent initialization was considered more appropriate for the assessments performed in this work, while parameter tracking represents a promising strategy to improve the efficiency of future practical applications of VQE.

Due to the computational time and memory required for AS8, UCCSD and GateFabric were evaluated using only one VQE run initialized with all variational parameters set to zero. In contrast, the AS8 results for *k*-UpCCGSD and GRSD correspond to the average of ten independent runs. Consequently, the relative performance of UCCSD and GateFabric compared to *k*-UpCCGSD and GRSD in AS8 should not be interpreted as a strictly equivalent statistical comparison.

## Results and discussion

In the NISQ era, the efficient use of resources is crucial, as unnecessary parameters hinder the scalability of quantum algorithms [1, 16]. Therefore, evaluating the effectiveness of VQE in computing molecular ground-state energies with different *ansätze*, initial parameters, and active spaces is essential, given that its performance is highly dependent on these choices. Therefore, such analyses were carried out dissociating H$_{2}$, H$_{3}^{+}$, and CH$_{5}^{+}$.

### H$_{2}$ and H$_3^+$: comparison between *ansätze*

**Fig. 6:**
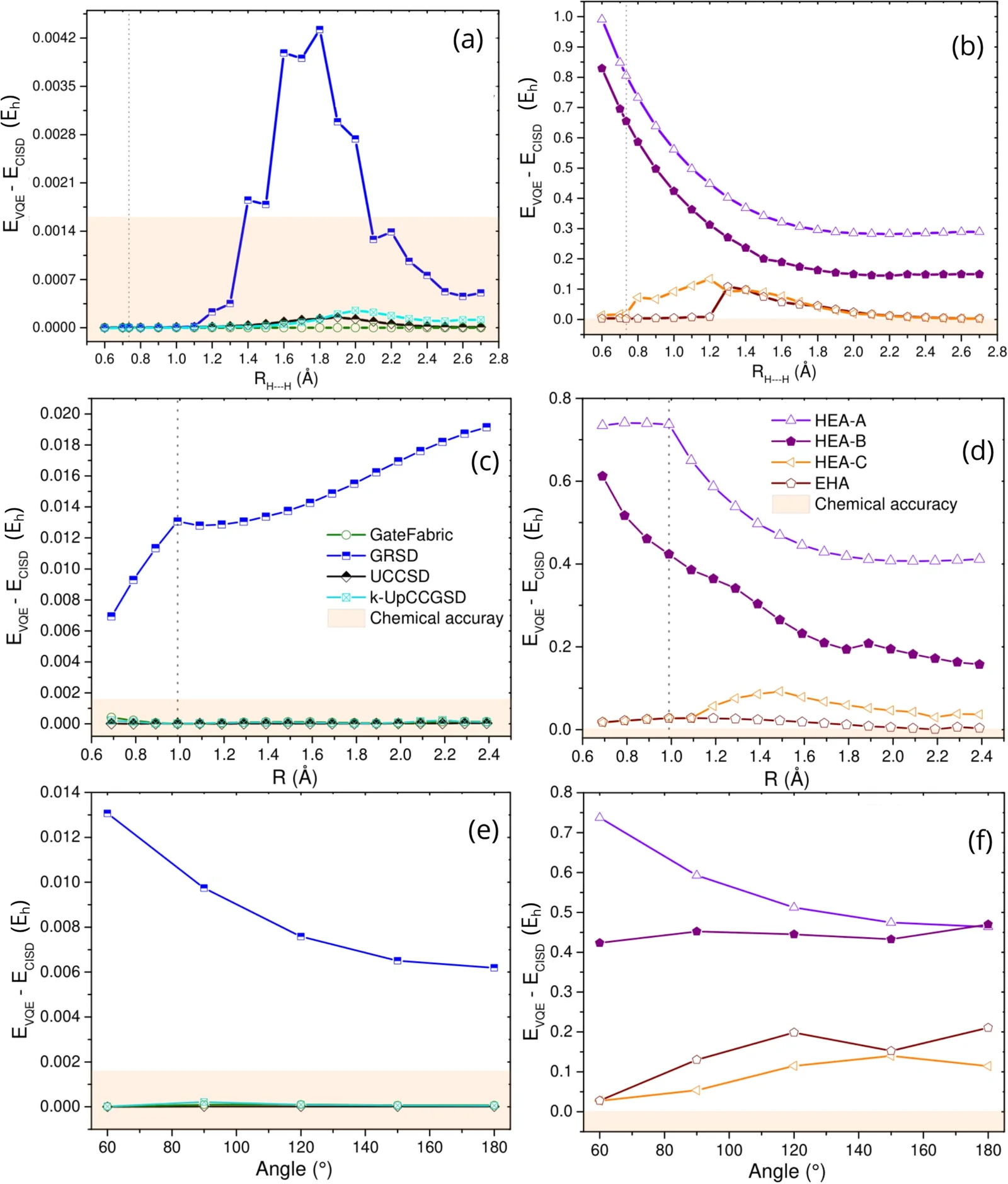
Average energy (in $E_h$) differences (from 10 VQE runs with distinct initial parameters), in $E_h$, along the dissociation coordinates of **a** and **b** H$_2$; **c** and **d** H$_3^+$ as a function of the interatomic distance (in Å); and **e** and **f** H$_3^+$ as a function of the angle variation (in degree), obtained with different VQE *ansätze* relative to CISD. The dashed vertical line indicates the equilibrium region of the molecules, while the shaded area represents the chemical accuracy region

Figure 6 shows the average differences (10 runs of the VQE with different initial parameters) in the energies of the equilibrium and dissociation geometries of H$_{2}$ and H$_{2}^+$ obtained with various *ansätze* compared to the CISD. The dissociation curves are available in the Supplementary Material (Figs. S1 and S2), while the average energy values in 10 runs of the VQE and the energies of the first run (with all variational parameters equal to zero) can be found in Tables S2, S3, S4, S5 and S6.

A brief comparison with high-precision data illustrates the physical limitations of the STO-3 G basis set. For H$_2$, near the equilibrium region, the CISD/STO-3 G energy obtained in this work was -1.13731 E$_h$ at R=0.73 Å. It is worth noting that, for this two-electron system, CISD is equivalent to FCI within a given orbital basis. The explicitly correlated Kolos-Wolniewicz approach provides a highly accurate description of the H$_2$ potential-energy curve and its dissociation process. Using this approach, a Born-Oppenheimer energy of approximately -1.174476 E$_h$ was reported at R=0.74 Å [60, 68]. Although the internuclear distances are not exactly identical, both values correspond to the near-equilibrium region, and the difference of approximately $3.72 \times 10^{-2}$ E$_h$ mainly reflects the limited orbital flexibility and basis-set incompleteness of STO-3 G. Thus, the comparison with the Kolos-Wolniewicz result assesses the limitations of the finite-basis molecular model, whereas the VQE-CISD difference assesses the performance of the variational circuits within that same model. From a practical perspective, extending VQE beyond minimal basis sets will likely require its combination with active-space techniques.

The *ansätze* that maintained the energy differences relative to CISD within chemical accuracy ($\le 1.6 \times 10^{-3}$ $E_h$ [34]) in all H${_2}$ and H$_3^+$ dissociation geometries were UCCSD, GateFabric, and *k*-UpCCGSD. The GRSD *ansatz* failed to achieve chemical accuracy for the dissociation geometries of H$_2$ at distances between 1.4 and 2.0 Å (Fig. 6a), while for H$_3^+$ this *ansatz* generated energies outside of chemical accuracy for all distances and angles evaluated. In Tables S2 and S4, it can be observed that, unlike the average of the 10 VQE runs with varying initial parameters, the runs with initial parameters equal to zero generated energies within chemical accuracy when using GRSD. This demonstrates that, when initializing the qubits in a state different from that obtained with Hartree-Fock occupation, GRSD has greater difficulty in obtaining the accurate energy of the ground state. Therefore, GRSD is highly sensitive to the choice of initial parameters.

Hardware-efficient *ansätze* generally exhibit significant deviations in the dissociation energies of H$_2$ and H$_3^+$ (Fig. 6b, d, and f). HEA-A and HEA-B [58] showed the largest energy differences relative to CISD. Moreover, these *ansätze* display a trend opposite to that of CISD: instead of increasing, the energy tends to decrease as H$_2$ or H$_3^+$ dissociates. For H$_2$, for example, the dissociation profiles obtained with HEA-A and HEA-B more closely resemble that of the excited state of this molecule [61]. Although HEA-C [58] does not achieve chemical accuracy for any of the geometries considered, it yields smaller errors relative to CISD than HEA-A and HEA-B around the equilibrium region of these molecules. However, HEA-C begins to show noticeable deviations for H$_2$ at 0.9 Å and for H$_3^+$ at 1.2 Å. Beyond 1.3 Å for H$_2$ and 1.6 Å for H$_3^+$, these discrepancies gradually decrease as the interatomic distance increases, although they remain above the chemical accuracy threshold.

HEA-A and HEA-B showed the largest energy differences. This can be attributed to the fact that HEA-A explores fewer excitation paths, as it applies Hadamard gates only to the highest-energy occupied molecular orbital (HOMO) and allows entanglement exclusively between HOMO and LUMO orbitals (Fig. 2a). In contrast, HEA-B and HEA-C apply Hadamard gates to all qubits and allow entanglement between HOMO-HOMO, LUMO-LUMO, and HOMO-LUMO, facilitating a wider range of excitations (Figs. 2 and 3). However, HEA-B exhibits low performance, possibly due to the use of a smaller number of RX and RY gates than HEA-C and EHA (Table 1). Notably, Kandala et al. [34] demonstrated that a different HEA successfully predicted the dissociation energy of H$_2$. This suggests that while some HEA may achieve high accuracy, others may yield inferior results depending on their design and parameterization.

For H$_3^+$, the energy obtained in the first VQE run, with all initial parameters set to zero, is higher than the average energy over the 10 runs (Fig. S2). For HEA-B and EHA-C, the first run also yields more positive energies, although the differences relative to the averages are smaller. This behavior is not observed for the chemically inspired *ansätze* and for the GateFabric. Similar trends are found in the analysis of the energy as a function of the angle (Fig. S2, and Tables S6 and S5).

EHA does not achieve chemical accuracy for the H$_3^+$ geometries, but it does reach chemical accuracy for H$_2$ at 2.19 Å, where the error is $1.06 \times 10^{-3}$ $E_h$. Even so, this *ansatz* yields energy deviations relative to CISD that are smaller than or comparable to those of HEA-C, and significantly smaller than those of HEA-A and HEA-B.

Wang et al. [66], who proposed EHA, evaluated this *ansatz* for the dissociation of H$_5$ and reported that its energy error is lower than that of ADAPT-VQE for all geometries considered. In the present work, however, the poorer performance of EHA is mainly associated with the use of different initial parameters over 10 VQE runs, which resulted in a large standard deviation and caused the average energy to deviate from the CISD reference (Fig. S2 and Table S2). When only the run with all initial parameters set to zero is considered for the H$_2$ geometries (Table S3) and H$_3^+$ geometries (Tables S5 and S6), EHA provides an excellent approximation to the CISD energy. These results show that, similarly to GRSD, EHA is strongly sensitive to the choice of initial parameters. Indeed, when initialized with all parameters set to zero, its energy approaches the CISD reference much more closely.

### CH$_{5}^{+}$: comparison between *ansätze* and the active space

**Fig. 7:**
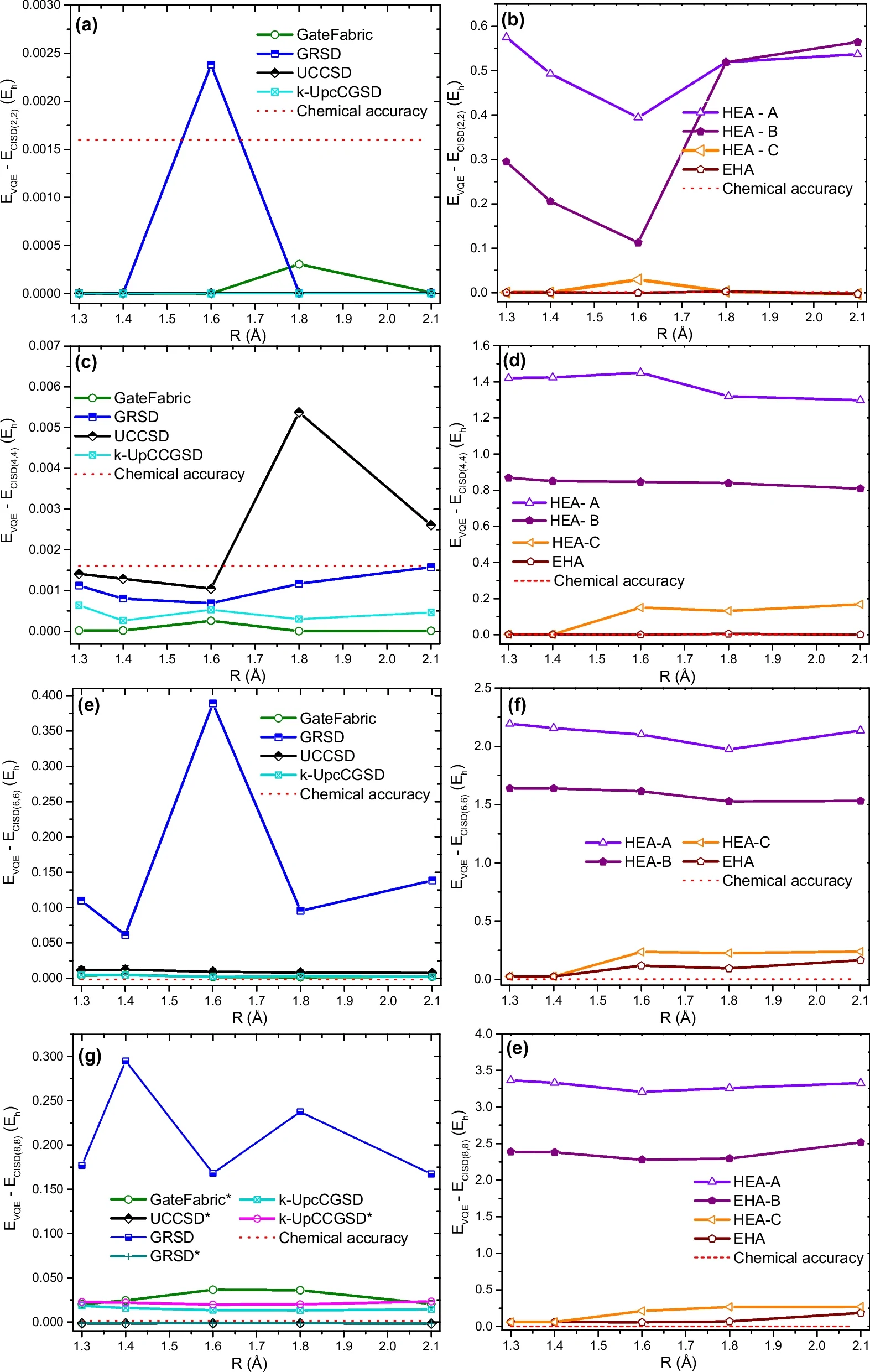
Differences energies (in $E_h$) of CH$_{5}^{+}$ dissociation geometries obtained with VQE using different *ansätze* and active spaces compared to CISD. The first, second, third, and fourth lines refer to active space (AS) 2, 4, 6, and 8, respectively. All results were obtained for 10 trials, except those indicated with an asterisk ∗ that consider all initial parameters as zero. The chemical accuracy region is represented by the area below the red dotted line

Compared to H$_2$ and H$_3^+$, CH$_5^+$ is a more challenging molecule due to its fluxional structure [67] and increasing multireferential behavior along the C–H$_2$ dissociation coordinate. Therefore, the performance of the different *ansätze* reflects not only their ability to reproduce the reference energies, but also their ability to represent the electronic rearrangement associated with the separation of the H$_2$ fragment. The average energy differences of 10 VQE runs, with different *ansätze*, in relation to CISD for the equilibrium and dissociation geometries of CH$_5^+$ are presented in Fig. 7, while the corresponding energies and the energies obtained with CASCI in the different active spaces tested in this work are shown in Fig. S3 and Tables S7 and S8.

As observed for H$_2$ and H$_3^+$, the *ansätze* that yielded results closest to CISD throughout the CH$_5^+$ dissociation profile were UCCSD, *k*-UpCCGSD, GRSD, and GateFabric (Fig. 7, Tables S7, S8, and S9). For AS2 (Fig. 7a), only GRSD at 1.6 Å failed to achieve chemical accuracy, with an error of $2.37 \times 10^{-3}$ $E_h$. For AS4 (Fig. 7c), only the energies of the last two dissociation geometries obtained with UCCSD remained outside the chemical accuracy threshold. For AS6, all four *ansätze* produced energies above the chemical accuracy limit for all CH$_5^+$ dissociation geometries. Among them, GateFabric and *k*-UpCCGSD yielded results closer to CISD, whereas GRSD showed the largest errors over the 10 VQE runs with distinct initial parameters. For those cases where chemical accuracy was not achieved, a high standard deviation in the energy of the 10 runs was observed, with these deviations being higher for GRSD. However, when analyzing the VQE executed only once, with the parameters set to zero, all circuits achieved chemical accuracy for all geometries in AS2, AS4, and AS6. (Table S7).

For AS8 (Fig. 7g), GateFabric and UCCSD were evaluated using only a single VQE run with all initial parameters set to zero, due to the computational time and memory limitations of the cluster employed in this work. Even when compared with the average over 10 runs, *k*-UpCCGSD outperformed GateFabric. However, neither of them achieved chemical accuracy. In contrast, GRSD and UCCSD, when evaluated in a single run, reached chemical accuracy for all CH$_5^+$ dissociation geometries.

**Table 1:** Counting logic gates and number of trainable parameters in different active spaces and *ansätze*, considering CH$_5^+$

AS	Ansatz	CNOT	H	RX	RY	RZ	Parameters
AS2	UCCSD	56	40	40	0	12	3
	GateFabric	380	0	542	120	1280	20
	*k*-UpCCGSD	128	0	162	0	184	6
	GRSD	18	0	12	12	20	3
	HEA-A	4	2	2	2	0	4
	HEA-B	3	2	2	2	0	4
	HEA-C	3	4	10	10	0	20
	EHA	15	0	15	4	14	21
AS4	UCCSD	896	608	608	0	160	26
	GateFabric	1140	0	1384	480	2280	60
	*k*-UpCCGSD	896	0	964	0	1104	36
	GRSD	268	0	128	160	248	26
	HEA-A	8	4	4	4	0	8
	HEA-B	7	4	4	4	0	8
	HEA-C	7	8	22	22	0	44
	EHA	35	0	35	8	30	45
AS6	UCCSD	4752	3168	3168	0	792	117
	GateFabric	1900	0	2306	800	3800	100
	*k*-UpCCGSD	2560	0	2406	0	2760	90
	GRSD	1422	0	636	828	1260	117
	HEA-A	12	6	6	6	0	12
	HEA-B	11	6	6	6	0	12
	HEA-C	11	12	34	34	0	68
	EHA	55	0	55	12	46	69
AS8	UCCSD	15,872	10,624	10,624	0	2688	360
	GateFabric	2660	0	3228	1120	5320	140
	*k*-UpCCGSD	5376	0	4488	5152	0	168
	GRSD	4656	0	2040	2688	4064	360
	HEA-A	16	8	8	8	0	16
	HEA-B	15	8	8	8	0	16
	HEA-C	15	16	46	46	0	92
	EHA	75	0	75	16	62	93

The hardware-efficient *ansätze* usually yielded lower energies when initialized with random parameters (Table S8). The smaller standard deviations of HEA-C and EHA indicate more consistent behavior across different initializations. The HEA-A and HEA-B exhibited the poorest performance, with deviations from the CISD reference increasing with the size of the active space for CH$_5^+$ for energy estimates, indicating that these circuits were not sufficiently expressive to describe the more complex electronic correlations introduced in AS6 and AS8. In contrast, HEA-C and EHA yielded average energies closer to CISD, particularly near equilibrium and at the first stretched geometry. Moreover, the energy deviations obtained with HEA-C and EHA varied less significantly with increasing active-space size than those obtained with HEA-A and HEA-B (Table S8 and Fig. S3). Nevertheless, the HEA-C and EHA energies remained close to the Hartree-Fock values, resulting in differences of approximately $6.0 \times 10^{-2}$ E$_h$ relative to CISD (Fig. 7). Thus, although HEA-C and EHA performed better than HEA-A and HEA-B, they introduced insufficient electronic correlation. This trend is also consistent with the study in which these circuits were proposed [58].

Regarding the success rate, defined as the number of VQE runs that achieved chemical accuracy among the ten independent runs, GRSD achieved chemical accuracy in only one for every geometry in both AS6 and AS8, while UCCSD showed the same success count in AS6. For GateFabric in AS6, no run achieved chemical accuracy at R=1.3 and 1.4 Å, whereas the number of successful runs increased to 3, 5, and 5 at R=1.6, 1.8, and 2.1 Å, respectively. Similarly, *k*-UpCCGSD showed no successful runs for the first three AS6 geometries, but achieved chemical accuracy in 6 of the 10 runs at both 1.8 and 2.1 Å. In AS8, none of the *k*-UpCCGSD runs reached chemical accuracy. These results demonstrate that the average performance may conceal isolated accurate solutions and further highlight the sensitivity of chemically inspired circuits to parameter initialization. Results for GateFabric and UCCSD in AS8 were not reported because all ten independent VQE runs could not be completed. Additionally, none of the hardware-efficient *ansätze* achieved chemical accuracy in any of the ten runs for any geometry in either AS6 or AS8.

It is also important to note that some *ansätze* may become more difficult to optimize as the active space increases, either because of the excessive number of trainable parameters or because of unfavorable optimization landscapes, such as barren plateaus. This effect is particularly evident for UCCSD and GRSD, whose number of trainable parameters increases the size of the active space sharply (Table 1). Both circuits contain only 3 parameters in AS2, whereas this number increases to 360 in AS8. A similarly pronounced growth is observed in the circuit size. UCCSD, for instance, requires 56 CNOT gates in AS2, but 15872 CNOT gates in AS8, representing a significant increase in circuit depth and implementation cost (Table 1). GRSD also shows substantial growth, reaching 4656 CNOT gates in AS8. It should also be noted that the number of trainable parameters is not necessarily equal to the number of rotation gates. This occurs particularly for some circuit templates implemented directly in the PennyLane library, in which certain rotations have fixed, non-optimized angles.

In contrast, GateFabric and *k*-UpCCGSD, which produced some of the most accurate CH$_5^+$ dissociation energies, exhibit a more moderate increase in both parameter count and logic gate count (Table 1). For GateFabric, there is an increase of 120 parameters when going from AS2 to AS8, while for *k*-UpCCGSD this increase is 162 parameters. Although these two approaches still generate relatively large circuits, their scaling is more favorable than that of UCCSD. For example, in AS8, GateFabric and *k*-UpCCGSD require 13212 and 10496 fewer CNOT gates, respectively, than UCCSD (Table 1). These results indicate that a larger number of parameters or logic gates does not necessarily translate into better performance.

On the other hand, the opposite extreme is also not advantageous. HEA-A and HEA-B contain very few parameters and very small gate counts in all active spaces, yet they consistently provide poor energy accuracy (Table 1). Thus, using fewer logic gates does not imply that a circuit will be less affected by optimization difficulties or that it will yield more accurate results. Rather, these results show that overly compact HEA may lack the expressibility required to describe the electronic structure with sufficient accuracy.

Therefore, the development of quantum circuits suitable for molecular energy calculations with VQE in the NISQ era requires a careful balance among circuit size, the scaling of circuit resources with the active-space dimension, and the parameter-initialization strategy. The difficulty of optimizing variational circuits may arise not only from the number of trainable parameters and quantum gates, but also from the region of the optimization landscape in which the initial state is placed. Inappropriate initialization or unphysical state mixing may drive the variational state toward sectors that do not represent the target electronic system, thereby compromising both the convergence of the optimization and the physical reliability of the resulting energy. Among the tested *ansätze*, GateFabric and *k*-UpCCGSD offered the best compromise between accuracy and scalability, as they achieve accuracy for dissociation energies, low sensitivity to initial parameters, and maintain a more moderate growth in the number of parameters and logic gates.

## Conclusions

The VQE was successfully applied to investigate the dissociation energies of H$_2$, H$_3^+$, and CH$_5^+$, comparing the results obtained with different *ansätze*, initial parameters, and active spaces to the CISD reference. The accuracy of VQE was found to depend strongly on the choice of *ansatz*. For the three molecular systems, the hardware-efficient *ansätze*, especially HEA-A and HEA-B, showed the largest deviations from CISD and were not adequate. For H$_2$ and H$_3^+$, UCCSD, GateFabric, and *k*-UpCCGSD provided the best overall results, remaining within chemical accuracy over the full dissociation profile.

For CH$_5^+$, the accuracy of VQE depends on both the *ansatz* and the active-space size. In general, increasing the active space led to larger energy errors, indicating that the *ansätze* may struggle to capture increasingly complex electronic correlation effects and may also face more difficult optimization conditions. Among the tested circuits, GateFabric and *k*-UpCCGSD provided the best balance between accuracy and scalability, yielding dissociation energies closer to CISD while showing a more moderate growth in the number of trainable parameters and logic gates. For the largest active space, UCCSD and GRSD achieved chemical accuracy only when evaluated in a single run with all initial parameters set to zero.

The results also show that GRSD and EHA are particularly sensitive to the choice of initial parameters. For H$_3^+$ and CH$_5^+$, averages over 10 VQE runs with different initial parameters may deviate substantially from CISD, whereas runs initialized with all parameters equal to zero can produce energies much closer to the reference. In contrast, UCCSD, GateFabric, and *k*-UpCCGSD showed a less pronounced dependence on initialization.

The analysis of trainable parameters and logic-gate counts indicates that neither a larger number of parameters nor a deeper circuit necessarily leads to better performance. Very large circuits, such as UCCSD and GRSD in larger active spaces, may become computationally expensive and more difficult to optimize, while very compact circuits, such as HEA-A and HEA-B, may not be expressive. Therefore, the efficiency of a variational circuit depends on a balance between expressibility, robustness to initialization, and circuit cost.

Overall, our results indicate that VQE can effectively describe the dissociation of small molecules with accuracy comparable to CISD under suitable conditions. However, achieving consistent chemical accuracy remains a challenge and requires careful selection of the *ansatz*, initial parameters, active space size, and optimization strategy. Future work will focus on evaluating other VQE strategies, particularly Quantum Architecture Search (QAS), to improve both accuracy and scalability, as well as performing simulations on real quantum computers with noise correction algorithms. In addition, the impact of alternative first- and second-order classical optimizers on the convergence behavior and hyperparameter tuning will be investigated, particularly for *ansätze* such as GRSD and HEA, which exhibited pronounced sensitivity to the initialization of the variational parameters.

## Supplementary Information

Below is the link to the electronic supplementary material.

Supplementary file 1 (pdf 343 KB)

## Data Availability

The data generated that support the findings of this study are available from the corresponding author upon reasonable request.

## Code availability

The code used in this study is available from the corresponding author upon reasonable request.
