# Supplementary material for: Assessment of the variational quantum eigensolver (VQE) for bond dissociation of H2, H3 + , and CH5 + 
Source: J Mol Model. 2026 Sep 25;32(10):385. doi: 10.1007/s00894-026-06951-w (PMC13615147; doi:10.1007/s00894-026-06951-w)
Supplement: Supplementary file 1 [file 894_2026_6951_MOESM1_ESM.pdf]

Supplementary Material for: Assessment of the Variational Quantum Eigensolver (VQE) for Bond Dissociation of H_2, H_3^+, and CH_5^+

Amanda Marques de Lima[1*†],
Eivson Darlivam Rodrigues de Aguiar Silva[1†],
Erico Souza Teixeira[2*†], Ricardo Luiz Longo[1†]

[1]Departamento de Química Fundamental, Universidade Federal de Pernambuco, Recife, PE, 50670-901, Brazil.
[2]Centro de Excelência em Computação Quântica, Venturus Centro de Inovação Tecnológica, Estrada Giuseppina Vianelli di Napolli, 1185, Campinas, SP, 13086-530, Brazil.

*Corresponding author(s). E-mail(s): amanda.aml002@gmail.com; teixeira.erico@gmail.com;
Contributing authors: eivsondras@gmail.com; ricardo.longo@ufpe.br;
†These authors contributed equally to this work.

Table S1 Number of single and double excitations using an active space with 2, 4, 6, and 8 electrons for CH_5^+.

**Number of excitations**	**2**	**4**	**6**	**8**
Single excitations	2	8	18	32
Double excitations	1	18	99	328

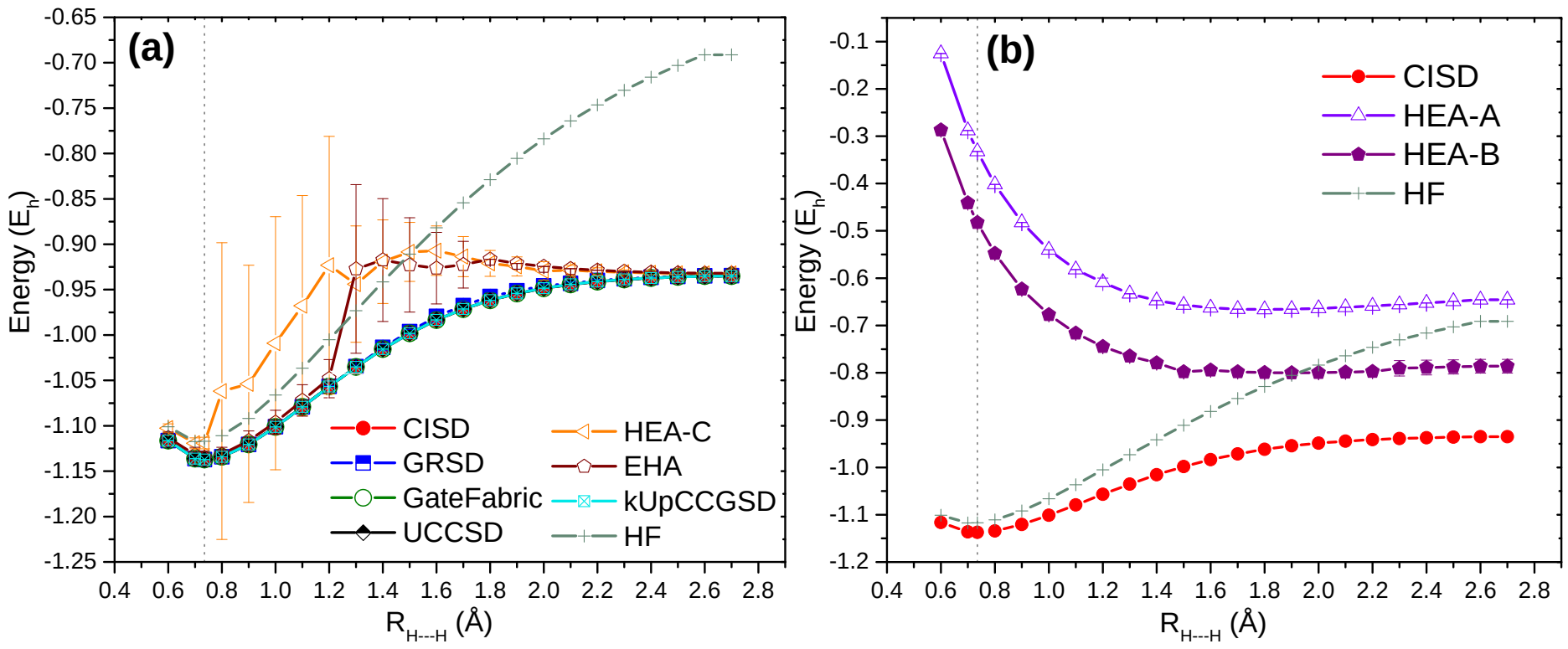

Fig. S1 Ground state energies (in E_h) of H_2 dissociation geometries obtained with the different *ansätze* as well as with Hartree-Fock (HF) and CISD. All VQE energies were averaged over 10 runs. For the chemically based *ansätze* and HEA-A and HEA-B, the standard deviations are generally smaller than 1×10^{-4} E_h.

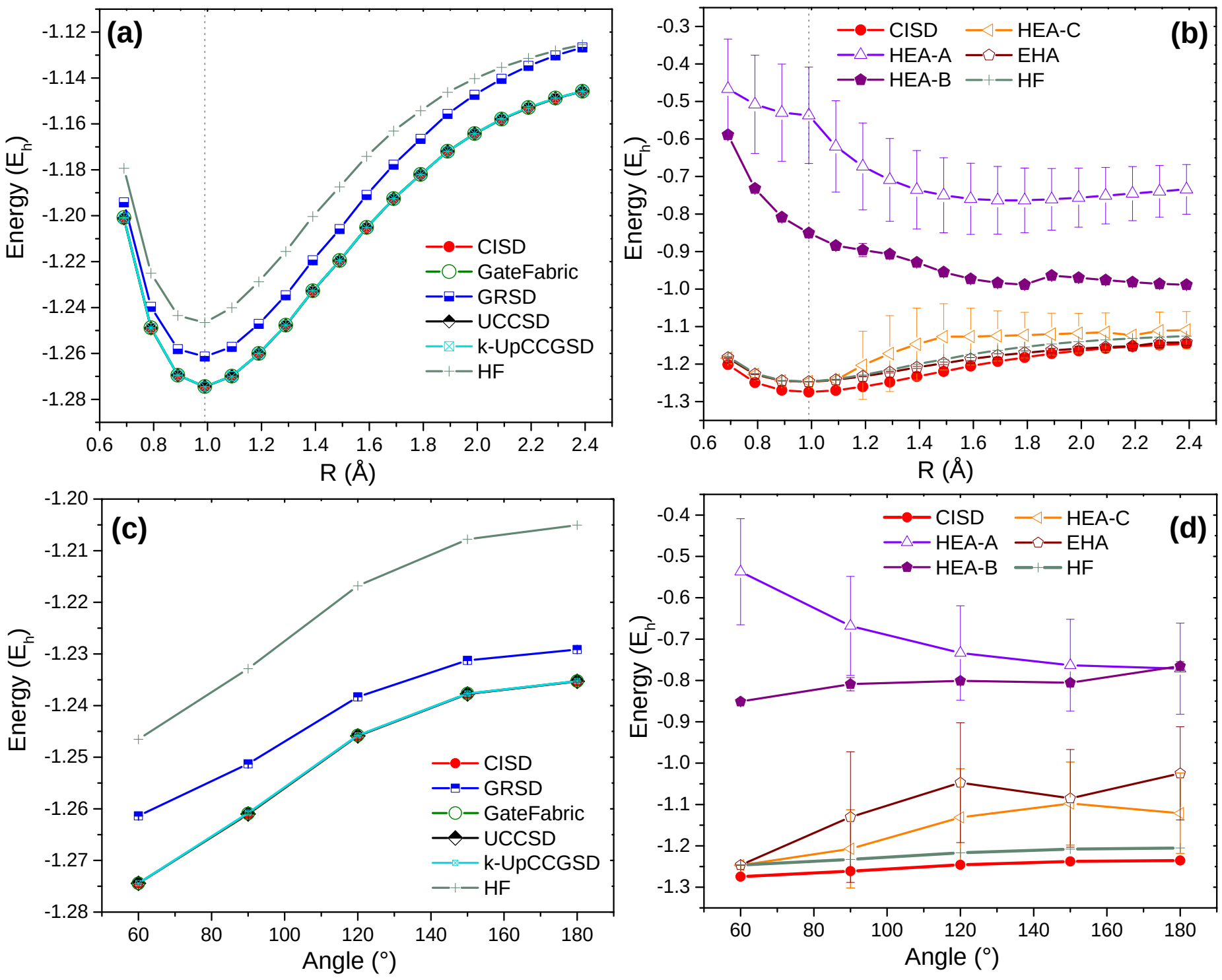

Fig. S2 Ground state energies of H_3^+ dissociation geometries for all *ansätze* and CISD as a function of distance (a-b) and angle (c-d). All VQE energies were obtained by averaging over 10 runs. For the chemically based *ansätze* the standard deviations are generally less than 1×10^{-4} E_h.

Table S2 Energies (in E_h) as a function of the dissociation distance (in Å) of H_2 dissociation geometries obtained using the chemistry-based *ansätze*. Columns with * indicate that only one trial was performed (Hartree-Fock), with all parameters equal to zero, while columns without $*$ indicate that 10 trials were performed.

R	CISD	GRSD*	GRSD	GateFabric*	GateFabric	UCCSD*	UCCSD	*k*-UpCCGSD*	*k*-UpCCGSD
0.6	-1.11629	-1.11629	-1.11628	-1.11629	-1.11629	-1.11629	-1.11628	-1.11629	-1.11629
0.7	-1.13619	-1.13619	-1.13619	-1.13619	-1.13619	-1.11629	-1.13619	-1.13619	-1.13619
0.73	-1.13731	-1.13731	-1.13730	-1.13731	-1.13731	-1.13731	-1.13730	-1.13730	-1.13730
0.8	-1.13415	-1.13415	-1.13414	-1.13415	-1.13415	-1.13415	-1.13414	-1.13413	-1.13415
0.9	-1.12056	-1.12056	-1.12056	-1.12056	-1.12056	-1.12056	-1.12055	-1.12056	-1.12056
1.0	-1.10115	-1.10115	-1.10114	-1.10115	-1.10115	-1.10115	-1.10114	-1.10115	-1.10115
1.1	-1.07919	-1.07919	-1.07918	-1.07919	-1.07919	-1.07919	-1.07918	-1.07918	-1.07919
1.2	-1.05674	-1.05674	-1.05651	-1.05674	-1.05674	-1.05674	-1.05672	-1.05674	-1.05673
1.3	-1.03519	-1.03518	-1.03483	-1.03519	-1.03519	-1.03518	-1.03516	-1.03518	-1.03517
1.4	-1.01547	-1.01547	-1.01362	-1.01547	-1.01547	-1.01547	-1.01543	-1.01547	-1.01545
1.5	-0.99815	-0.99815	-0.99636	-0.99815	-0.99815	-0.99815	-0.99809	-0.99815	-0.99812
1.6	-0.98347	-0.98347	-0.97949	-0.98347	-0.98347	-0.98347	-0.98339	-0.98347	-0.98342
1.7	-0.97143	-0.97142	-0.96752	-0.97143	-0.97143	-0.97142	-0.97131	-0.97142	-0.97135
1.8	-0.96182	-0.96181	-0.95750	-0.96182	-0.96182	-0.96181	-0.96169	-0.96181	-0.96169
1.9	-0.95434	-0.95434	-0.95135	-0.95434	-0.95434	-0.95434	-0.95419	-0.95433	-0.95414
2.0	-0.94864	-0.94864	-0.94590	-0.94864	-0.94864	-0.94864	-0.94852	-0.94864	-0.94839
2.1	-0.94437	-0.94437	-0.94309	-0.94437	-0.94437	-0.94437	-0.94428	-0.94437	-0.94415
2.2	-0.94122	-0.94122	-0.93983	-0.94122	-0.94122	-0.94122	-0.94117	-0.94122	-0.94105
2.3	-0.93892	-0.93892	-0.93796	-0.93892	-0.93892	-0.93892	-0.93889	-0.93892	-0.93879
2.4	-0.93725	-0.93725	-0.93650	-0.93725	-0.93725	-0.93725	-0.93724	-0.93725	-0.93715
2.5	-0.93605	-0.93605	-0.93553	-0.93605	-0.93605	-0.93605	-0.93604	-0.93605	-0.93596
2.6	-0.93520	-0.93519	-0.93474	-0.93519	-0.93519	-0.93519	-0.93519	-0.93519	-0.93508
2.7	-0.93410	-0.93519	-0.93469	-0.93519	-0.93519	-0.93519	-0.93519	-0.93519	-0.93508

Table S3 Energies (in E_h) as a function of the dissociation distance (in Å) of H_2 obtained using the HEA. Columns with * indicate that only one trial was performed, with all parameters equal to zero, while columns without * indicate that 10 trials were performed.

R	CISD	HEA-A*	HEA-A	HEA-B*	HEA-B	HEA-C*	HEA-C	EHA*	EHA
0.6	-1.11629	-0.12521	-0.12516	-0.28645	-0.28698	-1.10109	-1.10261	-1.10113	-1.11312
0.7	-1.13619	-0.28786	-0.28794	-0.44080	-0.44079	-1.11730	-1.11919	-1.11735	-1.13190
0.73	-1.13731	-0.33225	-0.33226	-0.48248	-0.48248	-1.11695	-1.11901	-1.11700	-1.13482
0.8	-1.13415	-0.40175	-0.40174	-0.54770	-0.54768	-1.11078	-1.06177	-1.11085	-1.13145
0.9	-1.12056	-0.48220	-0.48231	-0.62311	-0.62312	-1.09178	-1.05383	-1.09191	-1.11639
1.0	-1.10115	-0.54010	-0.54006	-0.67722	-0.67722	-1.06604	-1.00899	-1.06610	-1.09608
1.1	-1.07919	-0.58169	-0.58174	-0.71637	-0.71638	-1.03650	-0.96768	-1.03653	-1.07203
1.2	-1.05674	-0.60896	-0.60896	-0.74468	-0.74469	-1.00507	-0.92309	-1.00509	-1.04806
1.3	-1.03519	-0.63278	-0.63286	-0.76458	-0.76490	-0.97308	-0.94390	-0.85523	-0.92713
1.4	-1.01547	-0.64785	-0.64737	-0.77898	-0.77898	-0.94143	-0.91912	-0.87542	-0.91737
1.5	-0.99815	-0.65655	-0.65686	-0.79803	-0.79802	-0.90090	-0.90841	-0.89058	-0.92278
1.6	-0.98347	-0.66254	-0.66256	-0.79450	-0.79451	-0.89214	-0.90704	-0.90190	-0.92631
1.7	-0.97143	-0.66550	-0.66545	-0.79802	-0.79802	-0.91029	-0.91361	-0.91033	-0.92248
1.8	-0.96182	-0.66657	-0.66629	-0.79972	-0.79971	-0.91651	-0.92102	-0.91657	-0.91649
1.9	-0.95434	-0.66563	-0.66565	-0.80012	-0.80012	-0.92098	-0.92441	-0.92116	-0.92108
2.0	-0.94864	-0.66385	-0.66396	-0.79963	-0.79965	-0.94860	-0.92929	-0.92453	-0.92444
2.1	-0.94437	-0.66161	-0.66157	-0.79862	-0.79860	-0.92689	-0.92866	-0.92699	-0.92690
2.2	-0.94122	-0.65871	-0.65873	-0.79711	-0.79720	-0.92875	-0.92995	-0.92877	-0.92867
2.3	-0.93892	-0.65565	-0.65561	-0.79045	-0.79047	-0.93004	-0.93087	-0.93005	-0.92995
2.4	-0.93725	-0.65234	-0.65235	-0.78902	-0.78901	-0.93097	-0.93153	-0.93097	-0.93087
2.5	-0.93605	-0.64908	-0.64905	-0.78756	-0.78754	-0.93163	-0.93200	-0.93163	-0.93153
2.6	-0.93520	-0.64578	-0.64577	-0.78612	-0.78611	-0.93209	-0.93230	-0.93210	-0.93199
2.7	-0.93530	-0.64575	-0.64577	-0.78611	-0.78611	-0.93209	-0.93230	-0.93211	-0.93199

Table S4 Energies (in E_h) as a function of the distance (in Å) between the H_3^+ atoms, obtained using the chemistry-based *ansätze*. Columns with * indicate that only one trial was performed, with all parameters equal to zero, while columns without * indicate that 10 trial were performed.

R	CISD	UCCSD*	UCCSD	GateFabric*	GateFabric	GRSD*	GRSD	*k*-UpCCGSD*	*k*-UpCCGSD
0.69	-1.20117	-1.20116	-1.20116	-1.20117	-1.20076	-1.19423	-1.19423	-1.20117	-1.20096
0.79	-1.24900	-1.24900	-1.24900	-1.24900	-1.24878	-1.23971	-1.23971	-1.24900	-1.24888
0.89	-1.26950	-1.26950	-1.26949	-1.26950	-1.26944	-1.25817	-1.25816	-1.26948	-1.26946
0.99	-1.27444	-1.27444	-1.27443	-1.27444	-1.27443	-1.26138	-1.26137	-1.27442	-1.27442
1.09	-1.26992	-1.26992	-1.26991	-1.26992	-1.26990	-1.25713	-1.25713	-1.26992	-1.26988
1.19	-1.26005	-1.26005	-1.26005	-1.26005	-1.25999	-1.24718	-1.24718	-1.26005	-1.25999
1.29	-1.24775	-1.24775	-1.24774	-1.24775	-1.24765	-1.23470	-1.23469	-1.24775	-1.24767
1.39	-1.23282	-1.23281	-1.23281	-1.23282	-1.23269	-1.21945	-1.21944	-1.23282	-1.23272
1.49	-1.21960	-1.21960	-1.21959	-1.21960	-1.21947	-1.20586	-1.20586	-1.21960	-1.21951
1.59	-1.20521	-1.20521	-1.20520	-1.20521	-1.20509	-1.19095	-1.19094	-1.20521	-1.20514
1.69	-1.19262	-1.19261	-1.19260	-1.19261	-1.19253	-1.17775	-1.17775	-1.19261	-1.19257
1.79	-1.18205	-1.18204	-1.18203	-1.18204	-1.18199	-1.16655	-1.16655	-1.18204	-1.18202
1.89	-1.17196	-1.17195	-1.17194	-1.17196	-1.17192	-1.15572	-1.15572	-1.17196	-1.17193
1.99	-1.16428	-1.16427	-1.16425	-1.16427	-1.16423	-1.14734	-1.14734	-1.16427	-1.16420
2.09	-1.15799	-1.15798	-1.15796	-1.15798	-1.15790	-1.14039	-1.14038	-1.15798	-1.15783
2.19	-1.15298	-1.15297	-1.15295	-1.15297	-1.15283	-1.13478	-1.13478	-1.15297	-1.15274
2.29	-1.14889	-1.14888	-1.14885	-1.14888	-1.14874	-1.13016	-1.13016	-1.14888	-1.14875
2.39	-1.14588	-1.14587	-1.14584	-1.14587	-1.14574	-1.12673	-1.12673	-1.14586	-1.14576

Table S5 Energies (in E_h) as a function of the distance (in Å) between the H_3^+ atoms, obtained using the HEA. Columns with * indicate that only one trial was performed, with all parameters equal to zero, while columns without * indicate that 10 trial were performed.

R	CISD	HEA-A*	HEA-A	HEA-B*	HEA-B	HEA-C*	HEA-C	EHA*	EHA
0.69	-1.20117	-0.06828	-0.51069	-0.58916	-0.58916	-1.18395	-1.18400	-1.17932	-1.18327
0.79	-1.24900	-0.11454	-0.55132	-0.73202	-0.73202	-1.22738	-1.22754	-1.22504	-1.22705
0.89	-1.26950	-0.14136	-0.57303	-0.80854	-0.80855	-1.24465	-1.24472	-1.24348	-1.24437
0.99	-1.27444	-0.15181	-0.57965	-0.85070	-0.8507	-1.2471	-1.24695	-1.24655	-1.24698
1.09	-1.26992	-0.2548	-0.66052	-0.88436	-0.88438	-1.24241	-1.24281	-1.24009	-1.24195
1.19	-1.26005	-0.32568	-0.71181	-0.90192	-0.90192	-1.23341	1.23354	-1.22878	-1.23269
1.29	-1.24775	-0.37776	-0.74577	-0.91025	-0.91065	-1.22252	-1.22346	-1.21557	-1.22160
1.39	-1.23282	-0.42133	-0.77027	-0.93078	-0.93095	-1.20981	-1.20989	-1.20032	-1.20865
1.49	-1.21960	-0.45027	-0.78334	-0.95484	-0.95484	-1.19910	-1.19998	-1.18746	-1.19779
1.59	-1.20521	-0.47513	-0.79116	-0.97304	-0.97304	-1.18805	-1.18889	-1.17415	-1.18654
1.69	-1.19262	-0.49277	-0.79357	-0.98354	-0.98354	-1.17895	-1.18015	-1.16308	-1.17725
1.79	-1.18205	-0.50494	-0.79231	-0.98812	-0.98812	-1.15045	-1.15341	-1.15427	-1.16997
1.89	-1.17196	-0.51461	-0.78812	-0.98864	-0.98864	-1.16549	-1.16698	-1.14623	-1.16344
1.99	-1.16428	-0.52088	-0.78259	-0.98604	-0.98604	-1.16089	-1.16213	-1.14026	-1.15871
2.09	-1.15799	-0.52553	-0.77618	-0.98153	-0.98153	-1.15721	-1.15818	-1.13539	-1.15493
2.19	-1.15298	-0.5292	-0.76951	-0.97605	-0.97605	-1.15428	-1.15497	-1.13143	-1.15190
2.29	-1.14889	-0.53256	-0.76262	-0.96992	-0.96992	-1.15184	-1.15219	-1.12808	-1.14305
2.39	-1.14588	-0.53564	-0.75634	-0.96407	-0.96407	-1.14999	-1.15014	-1.12549	-1.14204

Table S6 Energies (in E_h) obtained as a function of the angle (in degrees) of H_3^+.

Angle	60	90	120	150	180
CISD	-1.27444	-1.26102	-1.24589	-1.23775	-1.23532
UCCSD*	-1.27444	-1.26102	-1.24589	-1.23775	-1.23532
UCCSD	-1.27443	-1.26101	-1.24588	-1.23774	-1.23531
GateFabric*	-1.27444	-1.26102	-1.24589	-1.23775	-1.23532
GateFabric	-1.27443	-1.26092	-1.24579	-1.23768	-1.23525
GRSD*	-1.26137	-1.25128	-1.23831	-1.23125	-1.22914
GRSD*	-1.26137	-1.25128	-1.23831	-1.23125	-1.22913
k-UpCCGSD*	-1.27442	-1.26102	-1.24589	-1.23775	-1.23532
k-UpCCGSD	-1.27442	-1.26081	-1.24580	-1.23769	-1.23527
HEA-A*	-0.15207	-0.30889	-0.39114	-0.39114	-0.42984
HEA-A	-0.53708	-0.66816	-0.73358	-0.76318	-0.77156
HEA-B*	-0.85086	-0.81420	-0.80102	-0.80529	-0.77217
HEA-B	-0.85086	-0.80889	-0.80103	-0.80529	-0.76514
HEA-C*	-1.24703	-1.23875	-0.98670	-1.01555	-1.02369
HEA-C	-1.24704	-1.20719	-1.13112	-1.09766	-1.12100
EHA*	-1.24592	-1.23286	-1.21680	-1.20780	-1.20504
EHA	-1.24642	-1.13044	-1.04718	-1.08510	-1.02470

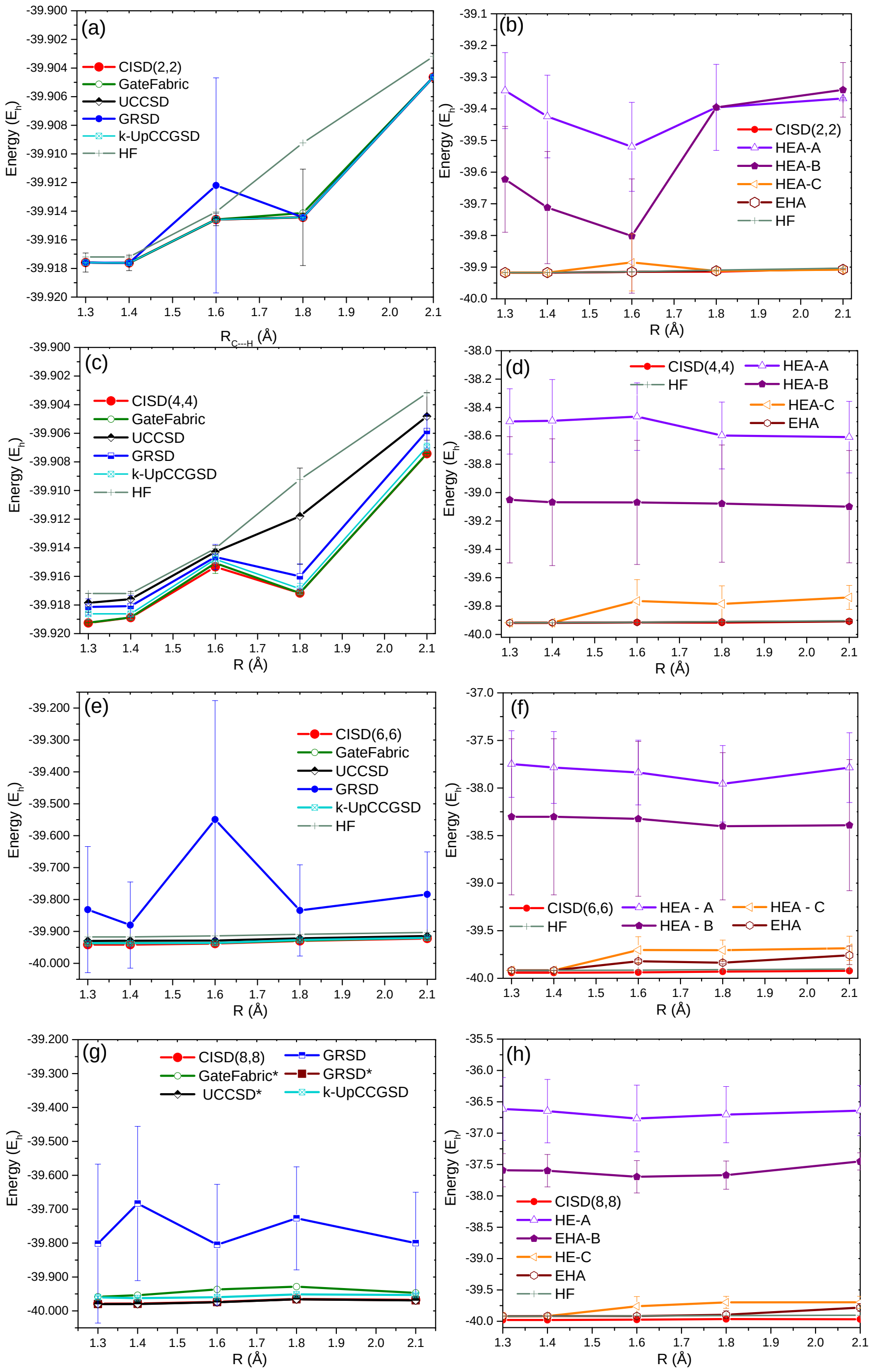

Fig. S3 Ground state energy of CH_5^+ for all *ansätze*, CISD and HF. First, second, third, and fourth rows of the panel refer to the AS2, AS4, AS6 and AS8, respectively. All VQE energies were obtained by averaging over 10 runs, except those indicated with $*$.

Table S7 Energies (in E_h) obtained by varying the distance (in Å) of CH_5^+ using CASCI and CISD in the active spaces ASn, where $n = 2$, 4, 6 and 8. Also presented are the average energies and standard deviations of 10 runs of the VQE with varying initial parameters, using chemistry-based *ansätze*. The symbols * indicate a single run of the VQE, in which all initial parameters were set to zero.

R	AS	CASCI	CISD	GRSD*	GRSD	GateFabric*	GateFabric	UCCSD*	UCCSD	k-UpCCGSD*	kUpCCGSD
1.3	2	-39.91759	-39.91759	-39.91759	$-39.91759 \pm 1 \times 10^{-4}$	-39.65779	$-39.91758 \pm 1 \times 10^{-5}$	-39.91759	$-39.91759 \pm 7 \times 10^{-4}$	-39.91759	$-39.91759 \pm 5 \times 10^{-7}$
1.4	2	-39.91762	-39.91762	-39.91762	$-39.91761 \pm 2 \times 10^{-4}$	-39.77277	$-39.91762 \pm 2 \times 10^{-6}$	-39.91762	$-39.91762 \pm 5 \times 10^{-4}$	-39.91762	$-39.91762 \pm 5 \times 10^{-7}$
1.6	2	-39.91458	-39.91458	-39.91458	$-39.91220 \pm 7 \times 10^{-3}$	-39.91458	$-39.91458 \pm 1 \times 10^{-6}$	-39.91458	$-39.91457 \pm 4 \times 10^{-4}$	-39.91458	$-39.91458 \pm 8 \times 10^{-7}$
1.8	2	-39.91444	-39.91444	-39.91443	$-39.91443 \pm 5 \times 10^{-7}$	-39.91444	$-39.91413 \pm 2 \times 10^{-6}$	-39.91443	$-39.91443 \pm 3 \times 10^{-3}$	-39.91443	$-39.91443 \pm 4 \times 10^{-7}$
2.1	2	-39.90463	-39.90463	-39.90463	$-39.90462 \pm 1 \times 10^{-5}$	-39.90463	$-39.90462 \pm 1 \times 10^{-6}$	-39.90463	$-39.90463 \pm 2 \times 10^{-3}$	-39.90463	$-39.90463 \pm 4 \times 10^{-7}$
1.3	4	-39.91926	-39.91926	-39.91926	$-39.91814 \pm 6 \times 10^{-4}$	-39.91926	$-39.91924 \pm 1 \times 10^{-5}$	-39.91926	$-39.91785 \pm 7 \times 10^{-4}$	-39.91925	$-39.91862 \pm 2 \times 10^{-4}$
1.4	4	-39.91889	-39.91888	-39.91888	$-39.91808 \pm 8 \times 10^{-4}$	-39.91888	$-39.91887 \pm 1 \times 10^{-5}$	-39.91888	$-39.91760 \pm 5 \times 10^{-4}$	-39.91888	$-39.91888 \pm 1 \times 10^{-3}$
1.6	4	-39.91534	-39.91533	-39.91533	$-39.91465 \pm 9 \times 10^{-4}$	-39.91533	$-39.91508 \pm 7 \times 10^{-4}$	-39.91533	$-39.91429 \pm 4 \times 10^{-4}$	-39.91533	$-39.91481 \pm 2 \times 10^{-4}$
1.8	4	-39.91720	-39.91717	-39.91719	$-39.91600 \pm 9 \times 10^{-4}$	-39.91709	$-39.91717 \pm 3 \times 10^{-5}$	-39.91719	$-39.91180 \pm 3 \times 10^{-3}$	-39.91710	$-39.91687 \pm 3 \times 10^{-3}$
2.1	4	-39.90743	-39.90743	-39.90743	$-39.90585 \pm 1 \times 10^{-3}$	-39.90743	$-39.90741 \pm 1 \times 10^{-5}$	-39.90743	$-39.90482 \pm 2 \times 10^{-3}$	-39.90742	$-39.90696 \pm 9 \times 10^{-4}$
1.3	6	-39.94179	-39.94150	-39.94163	$-39.83166 \pm 2 \times 10^{-1}$	-39.93715	$-39.93778 \pm 6 \times 10^{-4}$	-39.94163	$-39.92983 \pm 4 \times 10^{-3}$	-39.93710	$-39.93672 \pm 2 \times 10^{-4}$
1.4	6	-39.94177	-39.94151	-39.94164	$-39.88007 \pm 1 \times 10^{-1}$	-39.93659	$-39.93646 \pm 7 \times 10^{-4}$	-39.94165	$-39.92935 \pm 6 \times 10^{-3}$	-39.93788	$-39.93683 \pm 1 \times 10^{-3}$
1.6	6	-39.93844	-39.93824	-39.93839	$-39.54920 \pm 4 \times 10^{-1}$	-39.93659	$-39.93643 \pm 2 \times 10^{-4}$	-39.93840	$-39.92881 \pm 4 \times 10^{-3}$	-39.93654	$-39.93624 \pm 2 \times 10^{-4}$
1.8	6	-39.92998	-39.92980	-39.92994	$-39.83431 \pm 1 \times 10^{-1}$	-39.92858	$-39.92845 \pm 3 \times 10^{-4}$	-39.92994	$-39.92168 \pm 4 \times 10^{-3}$	-39.92702	$-39.92678 \pm 3 \times 10^{-3}$
2.1	6	-39.92246	-39.92222	-39.92236	$-39.78388 \pm 1 \times 10^{-1}$	-39.92106	$-39.92001 \pm 9 \times 10^{-4}$	-39.92232	$-39.91445 \pm 3 \times 10^{-3}$	-39.92011	$-39.92002 \pm 9 \times 10^{-4}$
1.3	8	-39.98062	-39.97864	-39.97970	$-39.80158 \pm 2 \times 10^{-1}$	-39.95839	–	-39.97998	–	-39.95581	$-39.96013 \pm 4 \times 10^{-3}$
1.4	8	-39.98029	-39.97832	-39.97939	$-39.68328 \pm 2 \times 10^{-1}$	-39.95385	–	-39.97966	–	-39.95624	$-39.96248 \pm 2 \times 10^{-3}$
1.6	8	-39.97509	-39.97315	-39.97420	$-39.80494 \pm 2 \times 10^{-1}$	-39.93659	–	-39.97442	–	-39.95324	$-39.95963 \pm 3 \times 10^{-3}$
1.8	8	-39.96636	-39.96455	-39.96569	$-39.72693 \pm 2 \times 10^{-1}$	-39.92858	–	-39.96571	–	-39.94443	$-39.95126 \pm 2 \times 10^{-3}$
2.1	8	-39.96979	-39.96741	-39.96901	$-39.79999 \pm 1 \times 10^{-1}$	-39.94678	–	-39.96910	–	-39.94406	$-39.95305 \pm 3 \times 10^{-3}$

Table S8 Average energies (in E_h) and standard deviations obtained by varying the distance CH_5^+ (in Å) using HEA and EHA, with n active spaces (ASn), with n = 2, 4, 6 and 8, in 10 trials. The symbol * indicates that the VQE was run only once, with all parameters set to zero.

R	AS	HEA-A*	HEA-A	HEA-B*	HEA-B	HEA-C*	HEA-C	EHA*	EHA
1.3	2	-38.95087	$-39.34256 \pm 1 \times 10^{-1}$	-39.14695	$-39.62266 \pm 2 \times 10^{-1}$	-39.91709	$-39.91711 \pm 3 \times 10^{-5}$	-39.91721	$-39.91715 \pm 2 \times 10^{-5}$
1.4	2	-38.98481	$-39.42439 \pm 1 \times 10^{-1}$	-39.17961	$-39.71196 \pm 2 \times 10^{-1}$	-39.91712	$-39.91714 \pm 2 \times 10^{-5}$	-39.91720	$-39.91715 \pm 1 \times 10^{-5}$
1.6	2	-39.06755	$-39.52008 \pm 1 \times 10^{-1}$	-39.25960	$-39.80187 \pm 2 \times 10^{-1}$	-39.91519	$-39.88514 \pm 3 \times 10^{-5}$	-39.91405	$-39.91504 \pm 3 \times 10^{-4}$
1.8	2	-39.07614	$-39.39542 \pm 1 \times 10^{-1}$	-39.07614	$-39.39542 \pm 2 \times 10^{-7}$	-39.91201	$-39.91204 \pm 1 \times 10^{-4}$	-39.90923	$-39.91165 \pm 8 \times 10^{-4}$
2.1	2	-39.09488	$-39.36748 \pm 2 \times 10^{-3}$	-39.09488	$-39.34022 \pm 9 \times 10^{-2}$	-39.90779	$-39.9078 \pm 8 \times 10^{-5}$	-39.90319	$-39.90727 \pm 1 \times 10^{-3}$
1.3	4	-37.81956	$-38.49830 \pm 2 \times 10^{-1}$	-37.79021	$-39.05080 \pm 4 \times 10^{-1}$	-39.91714	$-39.91711 \pm 4 \times 10^{-5}$	-39.91720	$-39.91715 \pm 2 \times 10^{-5}$
1.4	4	-37.81780	$-38.49425 \pm 3 \times 10^{-1}$	-37.79793	$-39.06778 \pm 4 \times 10^{-1}$	-39.91723	$-39.91714 \pm 5 \times 10^{-5}$	-39.91720	$-39.91715 \pm 2 \times 10^{-5}$
1.6	4	-37.79453	$-38.46425 \pm 2 \times 10^{-1}$	-37.8253	$-39.06897 \pm 4 \times 10^{-1}$	-39.91556	$-39.76445 \pm 2 \times 10^{-1}$	-39.91405	$-39.91498 \pm 3 \times 10^{-4}$
1.8	4	-37.93726	$-38.59744 \pm 2 \times 10^{-1}$	-37.91120	$-39.07749 \pm 4 \times 10^{-1}$	-39.64162	$-39.78515 \pm 2 \times 10^{-1}$	-39.90923	$-39.91159 \pm 8 \times 10^{-4}$
2.1	4	-37.96967	$-38.60905 \pm 2 \times 10^{-1}$	-37.98468	$-39.09903 \pm 4 \times 10^{-1}$	-39.69647	$-39.73872 \pm 8 \times 10^{-2}$	-39.90319	$-39.90717 \pm 1 \times 10^{-3}$
1.3	6	-37.85842	$-37.74782 \pm 3 \times 10^{-1}$	-35.97934	$-38.30259 \pm 8 \times 10^{-1}$	-39.91714	$-39.91712 \pm 4 \times 10^{-5}$	-39.91720	$-39.91716 \pm 2 \times 10^{-5}$
1.4	6	-36.76531	$-37.78441 \pm 4 \times 10^{-1}$	-36.01892	$-38.30259 \pm 8 \times 10^{-1}$	-39.91715	$-39.91714 \pm 2 \times 10^{-5}$	-39.91720	$-39.91717 \pm 1 \times 10^{-5}$
1.6	6	-36.86913	$-37.83766 \pm 3 \times 10^{-1}$	-36.01892	$-38.32413 \pm 8 \times 10^{-1}$	-39.61314	$-39.70248 \pm 1 \times 10^{-1}$	-39.91405	$-39.82049 \pm 1 \times 10^{-2}$
1.8	6	-36.74291	$-37.78578 \pm 4 \times 10^{-1}$	-36.20057	$-38.39052 \pm 8 \times 10^{-1}$	-39.66128	$-39.70416 \pm 1 \times 10^{-1}$	-39.90923	$-39.83640 \pm 1 \times 10^{-2}$
2.1	6	-36.74291	$-37.78578 \pm 4 \times 10^{-1}$	-38.39052	$-38.39052 \pm 8 \times 10^{-7}$	-39.69658	$-39.68419 \pm 1 \times 10^{-1}$	-39.90319	$-39.75784 \pm 1 \times 10^{-1}$
1.3	8	-35.18655	$-36.61444 \pm 5 \times 10^{-1}$	-36.91533	$-37.59083 \pm 3 \times 10^{-1}$	-39.91713	$-39.91716 \pm 9 \times 10^{-5}$	-39.91720	$-39.91716 \pm 2 \times 10^{-5}$
1.4	8	-35.21067	$-36.64819 \pm 5 \times 10^{-1}$	-36.93543	$-37.59821 \pm 3 \times 10^{-1}$	-39.91714	$-39.91715 \pm 3 \times 10^{-5}$	-39.91720	$-39.91665 \pm 2 \times 10^{-5}$
1.6	8	-35.25118	$-36.76621 \pm 5 \times 10^{-1}$	-37.02597	$-37.69482 \pm 3 \times 10^{-1}$	-39.61593	$-39.76043 \pm 2 \times 10^{-1}$	-39.91405	$-39.91585 \pm 2 \times 10^{-5}$
1.8	8	-35.42955	$-36.70452 \pm 4 \times 10^{-1}$	-37.12172	$-37.66940 \pm 2 \times 10^{-1}$	-39.66047	$-39.69754 \pm 9 \times 10^{-2}$	-39.90923	$-39.89575 \pm 6 \times 10^{-2}$
2.1	8	-35.27462	$-36.63993 \pm 4 \times 10^{-1}$	-37.25049	$-37.45034 \pm 1 \times 10^{-1}$	-39.69773	$-39.69658 \pm 1 \times 10^{-1}$	-39.90319	$-39.78075 \pm 6 \times 10^{-2}$

Table S9 Minimum energies, in E_h, obtained among independent VQE runs for CH_5^+ using different *ansätze* in the AS6 and AS8 active spaces. CISD energies obtained in the same active spaces are also reported. The symbol * indicates that all ten VQE runs could not be performed.

Ansatz	**1.3**	**1.4**	**1.6**	**1.8**	**2.1**
		**AS6**			
CISD	-39.94150	-39.94151	-39.93824	-39.92980	-39.92222
GRSD	-39.94163	-39.94164	-39.93839	-39.92994	-39.92236
GateFabric	-39.93912	-39.93911	-39.93665	-39.92899	-39.92115
UCCSD	-39.94163	-39.94165	-39.93840	-39.92994	-39.92223
k-UpCCGSD	-39.93710	-39.93788	-39.93654	-39.92858	-39.92082
HEA-A	-37.85842	-37.89765	-37.94527	-38.08299	-37.90165
HEA-B	-38.62050	-38.64983	-38.69641	-38.50215	-38.52365
HEA-C	-39.91717	-39.91715	-39.91522	-39.91215	-39.90744
EHA	-39.91720	-39.91720	-39.91522	-39.91191	-39.90774
		**AS8**			
CISD	-39.97864	-39.97832	-39.97315	-39.96455	-39.96741
GRSD	-39.97970	-39.97939	-39.97420	-39.96569	-39.96901
GateFabric*	-39.95839	-39.95385	-39.93659	-39.92858	-39.94678
UCCSD*	-39.97998	-39.97966	-39.97442	-39.96571	-39.9691
k-UpCCGSD	-39.96307	-39.96360	-39.96111	-39.95238	-39.95442
HEA-A	-36.77310	-36.80791	-36.93455	-36.84618	-36.79163
HEA-B	-37.77382	-37.78952	-37.85672	-37.87857	-37.64948
HEA-C	-39.91742	-39.91715	-39.91518	-39.91235	-39.94850
EHA	-39.91718	-39.91720	-39.91523	-39.91201	-39.90319
